# STING agonism enhances anti-tumor immune responses and therapeutic efficacy of PARP inhibition in *BRCA*-associated breast cancer

**DOI:** 10.1038/s41523-022-00471-5

**Published:** 2022-09-06

**Authors:** Constantia Pantelidou, Heta Jadhav, Aditi Kothari, Renyan Liu, Gerburg M. Wulf, Jennifer L. Guerriero, Geoffrey I. Shapiro

**Affiliations:** 1 Department of Medical Oncology, Dana-Farber Cancer Institute, Boston, MA USA; 2 Department of Medicine, Division of Hematology-Oncology and Cancer Research Institute, Beth Israel Deaconess Medical Center and Harvard Medical School, Boston, Massachusetts USA; 3 Ludwig Center for Cancer Research at Harvard, Harvard Medical School, Boston, MA USA; 4 Breast Tumor Immunology Laboratory, Department of Cancer Biology, Dana-Farber Cancer Institute, Boston, MA USA; 5 Division of Breast Surgery, Department of Surgery, Brigham and Women’s Hospital, Boston, MA USA; 6 Department of Medicine, Brigham and Women’s Hospital and Harvard Medical School, Boston, MA USA; 7 Present Address: Bayer Pharmaceuticals, Cambridge, MA USA

**Keywords:** Cancer immunotherapy, Breast cancer, Targeted therapies

## Abstract

Poly (ADP-ribose) polymerase (PARP) inhibitors exert their efficacy via synthetic lethal effects and by inducing cGAS/STING-mediated immune responses. We demonstrate that compared to monotherapies, combined PARP inhibition and STING agonism results in increased STING pathway activation, greater cytotoxic T-cell recruitment and enhanced dendritic cell activation in BRCA1-deficient breast cancer models. The combination markedly improved anti-tumor efficacy in vivo, with evidence of complete tumor clearance, prolongation of survival and induction of immunologic memory.

## Introduction

PARP inhibitors (PARPi) have improved treatment outcomes of *BRCA*-associated breast cancer (BC) and of other homologous recombination (HR) repair-deficient cancers^1^. In addition to mechanisms underlying the synthetic lethality of PARP inhibition and HR deficiency^2^, PARPi induce innate immune responses via cGAS/STING pathway activation, resulting in cytotoxic T-cell infiltration, an event critical for maximal efficacy in BRCA-deficient models^3,4^. These results have prompted the initiation of clinical trials combining PARP inhibition with immune checkpoint blockade, with the goal of further activating T-cell responses and overcoming PARPi resistance^5,6^. However, preliminary data from these trials have suggested that PD-1/L1 blockade may not enhance efficacy over PARP inhibition alone^6^. Recent results have indicated that PARP inhibition may also result in recruitment of immune suppressive macrophages and that PARPi-mediated T-cell activation can be improved with macrophage-targeting strategies^7^. Here, we have investigated an alternative approach to improving efficacy in *BRCA*-associated BC by combining PARP inhibition with STING agonism. STING agonists have entered clinical trials and combinations with chemotherapy or immune checkpoint blockade have demonstrated preliminary safety and efficacy^8,9^.

To determine whether pharmacological activation of the cGAS/STING pathway would further augment PARPi-induced inflammatory signaling, we combined olaparib and the STING agonist ADU-S100^10,11^, which has been studied in clinical trials^8,12,13^. Immunoblot analysis of phosphorylated STING and its effector TBK1 in KB1P-G3 cells derived from the K14-Cre-*Brca1*^*f/f*^*;Trp53*^*f/f*^ genetically engineered mouse model (GEMM) of triple-negative BC (TNBC), demonstrated activation of STING-TBK1 signaling in response to olaparib or ADU-S100 that was enhanced by combination treatment (Supplementary Fig. 1). Similar results were obtained in the human *BRCA1*-mutant TNBC cell line MDA-MB-436 and the *BRCA1/BRCA2*-mutant cell line HCC1395 (Supplementary Figs. 2 and 3). In these cell lines, the activation of STING signaling resulted in the production of β-interferon (IFNβ) (Supplementary Figs. 1b, 2b and 3b) and T cell-attracting chemokines, such as CCL5 and CXCL10 (Supplementary Figs. 1c, d, 2c, d and 3c, d), with greater effects of combination treatment compared to monotherapies at 24 hours after exposure. STING pathway activation did not occur in response to olaparib or the combination in MDA-MB-436 cells in which BRCA1 expression was repleted^3^ (Supplementary Fig. 2e, f).

**Fig. 1:**
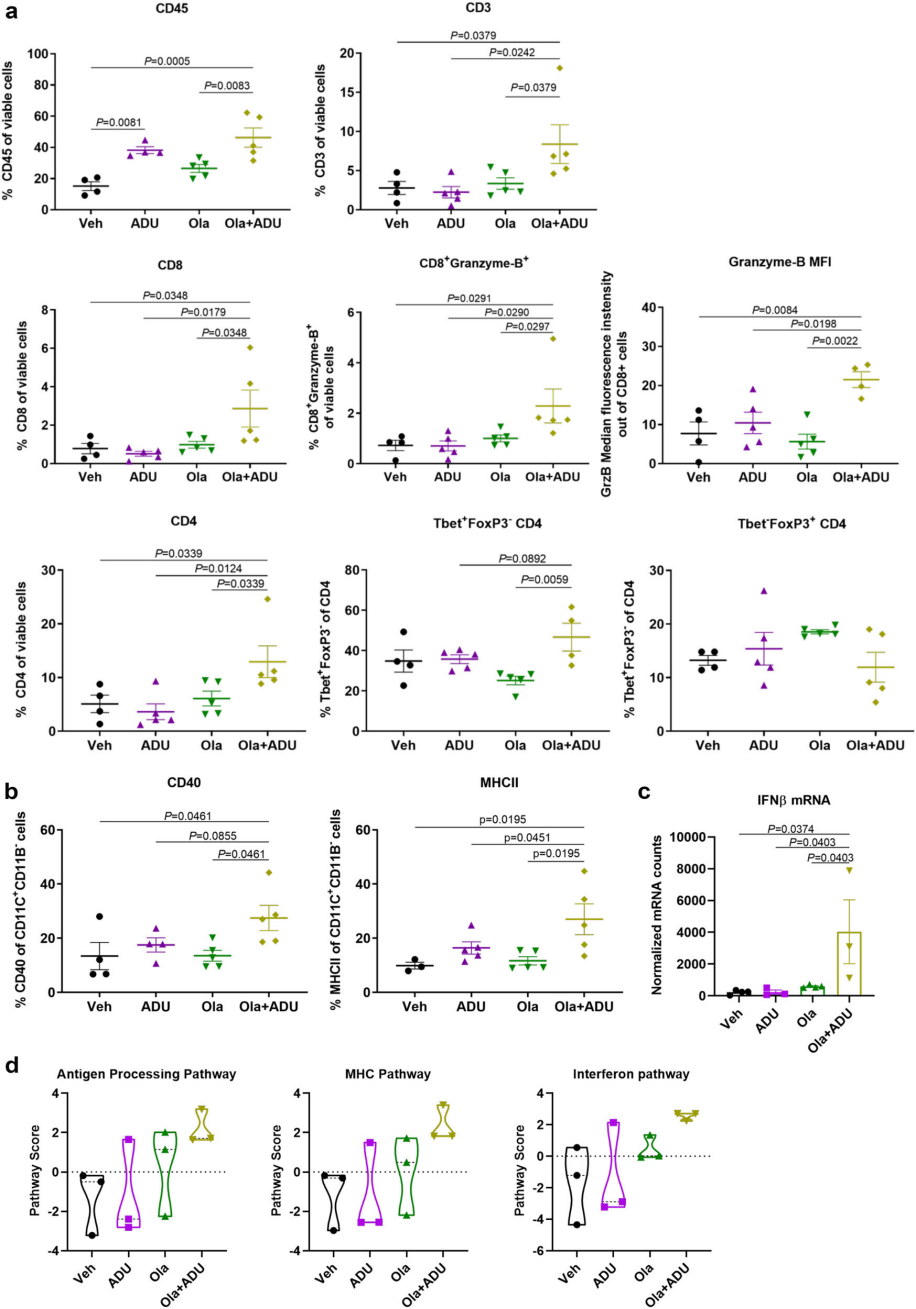
STING agonism and PARP inhibition cooperate to induce anti-tumor immune responses in BRCA-deficient TNBC tumors. Tumor chunks from the K14-Cre*-Brca1*^*f/f*^*;Trp53*^*f/f*^ GEMM were transplanted in syngeneic FVB/129 P mice, with 4-5 mice/ treatment group. IP injections were administered daily and intratumoral injections on day 1. **a**, **b** At 3 days, tumors were harvested, and single-cell suspensions subjected to flow cytometry. Scatter plots show (**a**) CD45^+^ cells, CD3^+^ cells, CD8^+^ and CD4^+^ T-cells, granzyme-B^+^ CD8 T-cells, granzyme-B median fluorescence intensity (MFI) in CD8 cells, Tbet^+^FoxP3^-^ T-helper 1 CD4 cells, Tbet^-^FoxP3^+^ T-regulatory CD4 cells, and (**b**) CD40^+^ and MHCII^+^ CD11C^+^CD11B^-^ dendritic cells. **c** At 7 days, tumors were harvested, and RNA isolated and subjected to qPCR analysis of IFNβ mRNA expression. Error bars are S.E.M. For (**a-c**), statistical analyses were performed using one-way ANOVA and Holm–Sidak post-hoc test. **d** Violin plot of the nanoString Pathway Scores in the top 3 upregulated pathways, summarizing data from a pathway’s genes with a single score.

We next sought to determine whether increased STING-TBK1 signaling in response to combined PARP inhibition and STING agonism in cancer cells translated to enhanced anti-tumor immune responses in vivo. To this end, we analyzed K14-Cre-*Brca1*^*f/f*^*;Trp53*^*f/f*^ breast tumors implanted in syngeneic mice and treated with olaparib, ADU-S100 or the combination, for the presence and activation of immune cells. Total leukocyte CD45^+^ counts were elevated in response to all treatments at 3 (Fig. 1a) and 7 days (Supplementary Fig. 4a, b). The combination of olaparib and ADU-S100 significantly increased total T-cell counts compared to monotherapies, with both CD8^+^ and CD4^+^ T-cell subsets significantly augmented (Fig. 1a). Enhanced T-cell recruitment in response to the olaparib/ADU-S100 combination was accompanied by activation of CD8^+^ T-cell cytolytic functions, as demonstrated by the significantly increased recruitment of granzyme-B^+^ CD8^+^ T-cells, as well as elevated granzyme-B total expression (Fig. 1a). In the case of CD4^+^ T-cells, the combination treatment increased T-helper 1 and not T-regulatory CD4^+^ T-cells, as measured by the expression of their respective markers, Tbet and FoxP3 transcription factors (Fig. 1a). Moreover, combining olaparib with ADU-S100 significantly increased the activation and antigen presentation capability of dendritic cells (DCs), as shown by the increases in CD40 and major histocompatibility (MHC) II expression in CD11C^+^CD11B^-^ DCs (Fig. 1b). In contrast to the combination therapy that led to a rapid induction of immune responses, olaparib monotherapy-induced T-cell recruitment and activation was more evident at 7 days (Supplementary Fig. 4a) compared to 3 days (Fig. 1a). Activated immune cell infiltration following ADU-S100 and olaparib treatment was accompanied by type-I IFN production, as evidenced by the significant increase in whole-tumor IFNβ mRNA levels compared to single treatments (Fig. 1c). Therefore, the addition of STING agonism to PARP inhibition elicits a superior immune response compared to monotherapies, characterized by increased cytotoxic T-cell recruitment and activation, and enhanced DC activation and antigen presentation.

To gain additional mechanistic insights into the anti-tumor immune response induced by combined PARP inhibition and STING agonism, we performed nanoString immune gene expression analysis in K14-Cre-*Brca1*^*f/f*^*;Trp53*^*f/f*^ tumors treated with olaparib, ADU-S100 or the combination. Gene set analysis revealed that the top-most upregulated genes in response to the olaparib/ADU-S100 combination were involved in antigen processing, MHC, interferon and leukocyte pathways (Supplementary Fig. 5a, Supplementary Table 1). The enrichment of these gene sets in response to combination therapy is also illustrated by the nanoString pathway scores (Fig. 1d). In addition to the top-most upregulated gene sets (as compared to vehicle treatment), dendritic cell functions and tumor necrosis factor (TNF) superfamily gene signatures were highly upregulated in comparison to olaparib and ADU-S100 alone (Supplementary Fig. 5a, Supplementary Table 1). Differential gene expression is shown by volcano plots (Supplementary Fig. 5b). Plotting of the normalized mRNA counts confirmed the significant increase in expression of 15 genes in response to the olaparib/ADU-S100 combination as compared to single treatments (Supplementary Fig. 6a). Among the top 5 most significantly induced genes were the H-2 class II histocompatibility antigen, A-B alpha (H2-Aa) and A-K beta (H2-Ab1) chains involved in antigen processing and interferon responses^14^; the C-type lectin domain family 7 member A (Clec7a) involved in innate responses, phagocytosis and leukocyte functions^15^; the TNF superfamily member Lymphotoxin-beta (Ltb) involved in cytotoxic T-cell effector functions^16^; and the integrin alpha L chain (Itgal) involved in leukocyte adhesion and mature T-cell functions^17,18^ (Supplementary Fig. 6a, Supplementary Table 2). The significant increases in mRNA expression of the top genes were validated by quantitative PCR analysis (Supplementary Fig. 6b). In summary, the mRNA expression analyses are consistent with the enhanced cytotoxic T-cell and DC activation observed by flow cytometry (Fig. 1, Supplementary Fig. 4) and demonstrate that the superior anti-tumor immune response observed upon combined PARP inhibition and STING agonism is a result of enhanced interferon signaling, antigen processing, and leukocyte and DC functions.

**Fig. 2:**
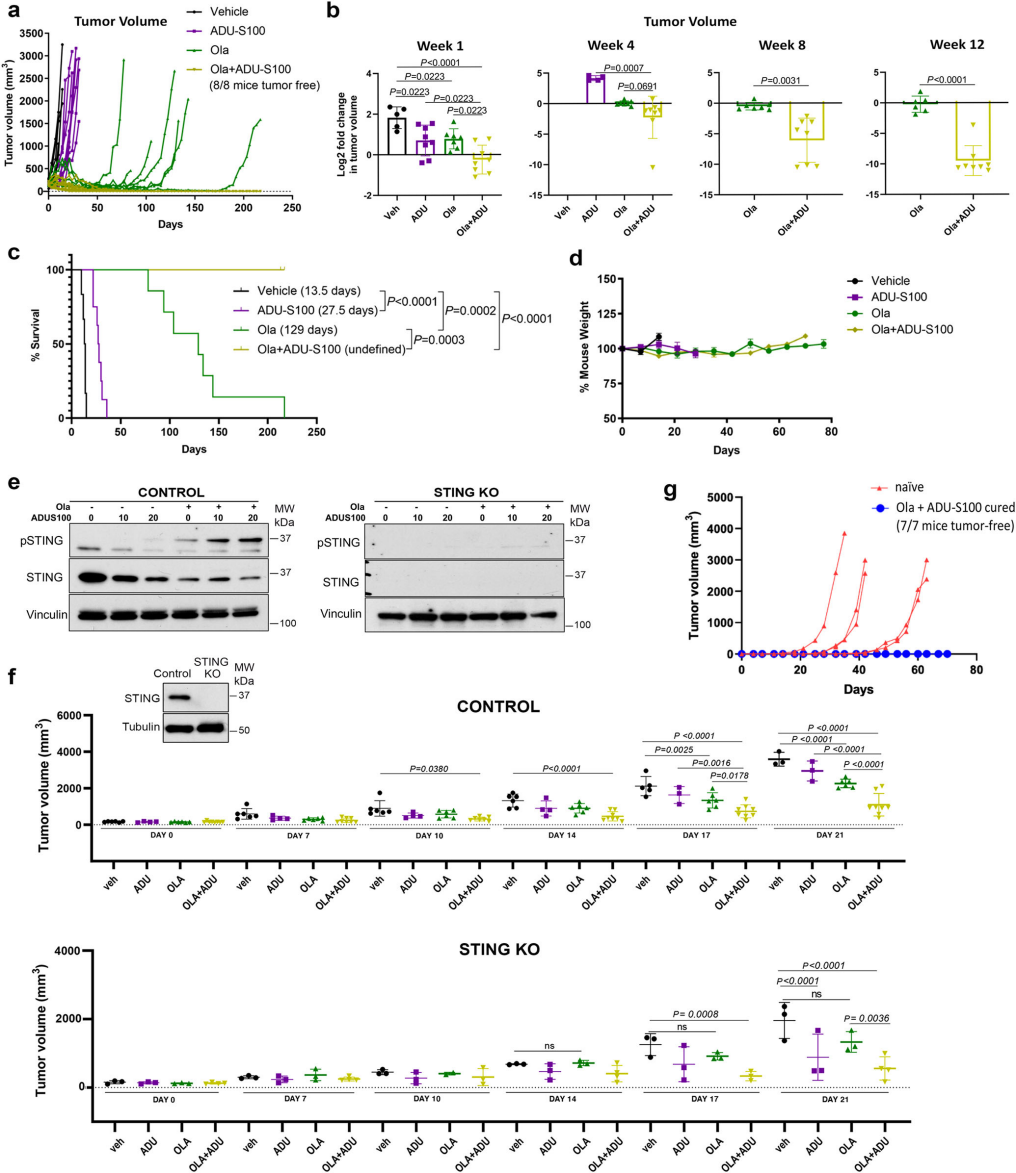
STING agonism potentiates the therapeutic efficacy of PARP inhibition in a BRCA-deficient model of TNBC, overcomes resistance and contributes to immunologic memory. Tumor chunks from the K14-Cre*-Brca1*^*f/f*^*;Trp53*^*f/f*^ GEMM were transplanted in syngeneic FVB/129P mice, which were treated with vehicle, olaparib (daily), ADU-S100 (weekly) or their combination (6–8 mice/group). Tumor volume was measured twice weekly and survival recorded. **a** Tumor volumes in individual mice over time. **b** Log2 fold-change in tumor volumes at weeks 1, 4, 8, 12. *P* values were determined using one-way ANOVA with Holm-Sidak post-hoc test at weeks 1 and 4 and with unpaired *t*-tests with Welch’s correction at weeks 8 and 12. **c** Percent survival. Median survival shown in brackets. Statistical analysis was performed using the Log-rank (Mantel–Cox) test. Intratumoral ADU-S100 injections were stopped when the tumor was cleared. **d** Weight of mice treated with vehicle, olaparib, ADU-S100 or their combination. **e** Murine KB1P-G3 CRISPR/Cas9 control or STING knockout (STING KO) cells were treated with vehicle (-, 0), 1 μM olaparib, the indicated doses of ADU-S100 (μg/ml) or their combination for 72 h and subjected to immunoblotting. **f** KB1P-G3 CRISPR/Cas9 control or STING KO tumors were transplanted in syngeneic FVB/129P2 mice, which were treated with vehicle, olaparib (daily), ADU-S100 (weekly) or their combination (4–8 mice/group). Tumor volumes (mm^3^) were measured twice weekly. Statistical analysis was performed using one-way ANOVA with Holm-Sidak post-hoc test. *(Inset)* Immunoblotting for total STING protein levels in KB1P-G3 control or STING KO tumors. **g** Tumor chunks were implanted in naïve mice or in the opposite mammary fat pads of those previously cured by combined olaparib/ADU-S100. Tumors could not be re-established in the latter mice.

To determine whether STING agonism can enhance the therapeutic efficacy of PARPi, we treated mice bearing K14-Cre-*Brca1*^*f/f*^*;Trp53*^*f/f*^ tumors with vehicle, olaparib, ADU-S100 or the combination. Treatment with olaparib resulted in tumor shrinkage and a median survival of 129 days (Fig. 2a–c). As expected, resistance to olaparib eventually emerged with most tumors relapsing after approximately 100 days of daily treatment (Fig. 2a). ADU-S100 alone showed modest therapeutic efficacy and doubled median survival to 27.5 days compared to vehicle control (Fig. 2a, c). The combination of ADU-S100 with olaparib resulted in significantly greater reduction in tumor volume than olaparib as early as week 1 (day 7), with most notable differences observed after week 8 (day 56) (Fig. 2a, b). Remarkably, the combination treatment led to complete tumor clearance in all enrolled mice (Fig. 2a) and 100% tumor-free survival across two experiments (Fig. 2c, Supplementary Fig. 7a, b). All treatments were well tolerated, and no animal weight loss was observed after long-term exposure (Fig. 2d). Furthermore, the olaparib/ADU-S100 combination was also efficacious against larger established tumors treated at a starting volume of 500-1,000 mm^3^ (Supplementary Fig. 8). As expected, efficacy of the combination was not observed when tumors were expanded in immunodeficient mice (Supplementary Fig. 9).

We previously demonstrated that intratumoral STING activation is required for full efficacy of olaparib in the K14-Cre-*Brca1*^*f/f*^*;Trp53*^*f/f*^ model. To assess the contribution of intratumoral STING pathway activation for combinatorial efficacy, we utilized control or STING knockout KB1P-G3 cells derived from a tumor from this model^3^. STING knockout was confirmed in engineered cells (Fig. 2e), as well as in orthotopically-established tumors (Fig. 2f, inset). Treatment of control cells confirmed the anti-tumor activity of olaparib and the improved efficacy of the olaparib/ADU-S100 combination through 21 days of treatment (Fig. 2f). In contrast, whereas the efficacy of olaparib monotherapy was compromised in mice bearing STING KO tumors, the combination was still efficacious in the absence of intratumoral STING, suggesting that STING agonism can favorably modulate the immune microenvironment to augment the activity of olaparib in an immunocompetent BRCA1-deficient BC model.

Finally, mice that achieved tumor clearance after combined STING agonism and PARP inhibition were rechallenged with tumor implantation. While tumors established in naïve mice, tumors could not be re-established in the opposite mammary fat pads of mice that had been cured (Fig. 2g). These findings demonstrate that STING agonism maximizes the anti-tumor efficacy of PARP inhibition, overcomes PARPi resistance and contributes to immunologic memory in BRCA-deficient TNBC models. Because the preclinical efficacy associated with intratumoral injection of a STING agonist may be difficult to translate to a heterogenous metastatic BC, it will be important to extend these findings to other tumor models and to determine whether similar results can be achieved with systemic agonists of STING, currently under development^19,20^. Nonetheless, the potent preclinical therapeutic efficacy of combined PARP inhibition and STING agonism warrants further development of this regimen as a treatment for *BRCA*-associated TNBC.

## Methods

### Cell culture

MDA-MB-436 and HCC1395 cells (ATCC) were verified with short tandem repeat profiling and along with MDA-MB-436 cells with reconstituted BRCA1 expression were maintained in RPMI supplemented with 10% FBS. The KB1P-G3 cell line was generated by seeding single-cell suspensions from breast tumors from K14-Cre-*Brca1*^*f/f*^;*Trp53*^*f/f*^ females, in DMEM (Corning) supplemented with 10% FBS, followed by serial passaging^3^. Cell lines were routinely tested for the presence of mycoplasma using the MycoAlert Mycoplasma Detection Kit (Lonza).

### Compounds

ADU-S100 (MIW815; Chemietek #CT-ADUS100) was reconstituted in USP normal saline (Thermo Fisher Scientific #NC9604723) and olaparib (Selleckchem for in vitro studies and MedChemExpress #HY-10162 for in vivo studies) was reconstituted in DMSO.

### Immunoblotting

Cells were lysed in RIPA buffer (Boston BioProducts) supplemented with protease and phosphatase inhibitors (Calbiochem) and quantitated for protein using the BCA Protein Assay Kit (Pierce). Equal amounts of protein were resolved by SDS-PAGE. Membranes were blocked in 5% milk/TBS-T (Boston BioProducts) and incubated with the following primary antibodies: phospho-TBK1/NAK (Ser172) (D52C2) XP Rabbit mAb (1:500) [Cell Signaling Technology (CST) #5483S] (for human cells), TBK1 (S172) (1:1000) (Abgent #AP7887a-ev) (for murine cells), TBK1/NAK (D1B4) Rabbit mAb (1:1000) (CST #3504S), phospho-STING (Ser366) (D7C3S) Rabbit mAb (1:1000) (CST #19781S) (for human cells), phospho-STING (Ser365) (D8F4W) Rabbit mAb (1:1000) (CST #72971S) (for murine cells), STING/TMEM173 (1:1000) (Novus Biologicals #NBP224683), Vinculin (1:1000) (CST #4650S). Immunodetection was performed using SuperSignal West Pico and Femto Chemiluminescent Substrate (Thermo Fisher Scientific). Blot stripping was performed using Restore PLUS Western Blot Stripping Buffer (Life Technologies) according to the manufacturer’s guidelines. Protein levels were quantified by densitometric analysis using ImageJ/Fiji. Phosphorylated protein levels were normalized to total protein band, then to loading control, and expressed as fold-change versus control DMSO. Uncropped and unprocessed blots are included in the Source Data files.

### Quantitative PCR

RNA was isolated, reverse transcribed and used for quantitative PCR. Primer sequences (5′–3′) were as follows: human IFNβ forward AACTTGCTTGGATTCCTACAAAG and reverse TATTCAAGCCTCCCATTCAATTG, mouse IFNβ forward CCAGCTCCAAGAAAGGACGA and reverse CGCCCTGTAGGTGAGGTTGAT, human CCL5 forward TGCCCACATCAAGGAGTATTT and reverse CTTTCGGGTGACAAAGACG, mouse CCL5 forward GCTCCAATCTTGCAGTCGTG and reverse GCTCCAATCTTGCAGTCGTG, human CXCL10 forward GGCCATCAAGAATTTACTGAAAGCA and reverse TCTGTGTGGTCCATCCTTGGAA, mouse CXCL10 forward CCAAGTGCTGCCGTCATTTT and reverse CTCAACACGTGGGCAGGATA, murine H2-Aa forward GACCTCCCAGAGACCAGGAT and reverse GGAACACAGTCGCTTGAGGA, murine Clec7a forward CCATAAAAGGCCCAGGGGAT and reverse TCGCCAAAATGCTAGGGCA, murine H2-Ab1 forward TGCTACTTCACCAACGGGAC and reverse TTTGCTCCAGGCAGACTCAG, murine Ltb forward GATGACAGCAAACCGTCGTG and reverse CAGCTGTTGAACCCCTGGAT, murine ItgaI forward TGGTCACTGAGCTGTCGTTC and reverse CTCAGGATAGGCTGCATGGC, human GAPDH forward GAGTCAACGGATTTGGTCGT and reverse TTGATTTTGGAGGGATCTCG, mouse GAPDH forward ACCACAGTCCATGCCATCAC and reverse TCCACCACCCTGTTGCTGTA.

### In vivo studies

All animal experiments were conducted in accordance with Institutional Animal Care and Use Committee-approved protocol #17-032. All mice were housed in a pathogen-free facility at Dana-Farber Cancer Institute. Tumors derived from the K14-Cre*-Brca1*^*f/f*^*;Trp53*^*f/f*^ TNBC mouse model, as well as KB1P-G3 control and STING KO cells, were transplanted into the mammary fat pad of FVB/129P2 or NSG mice (*NOD-Prkdc*^*em26Cd52*^*Il2rg*^*em26Cd22*^*/NjuCrl*). For efficacy studies, treatments were typically started once tumors reached 150-180 mm^3^ in volume and continued until tumors reached 20 mm in any direction, at which point mice were euthanized. In one experiment, treatments were started when tumors were between 500-1000 mm^3^ in volume. For flow cytometry studies, mice bearing tumors of 150-300 mm^3^ in volume were randomized in treatment groups, so that the average tumor volume in each group was the same. Mice received DMSO or DMSO-reconstituted olaparib, each diluted in saline immediately before intraperitoneal injection, with olaparib administered at 50 mg/kg daily. Vehicle (saline) or ADU-S100 was administered intratumorally weekly in a single 40 μl injection with ADU-S100 injections containing 50 μg compound. Therefore, in 4-arm experiments, mice were treated with vehicle (IP DMSO diluted in saline and intratumoral saline), IP olaparib (+ intratumoral saline), intratumoral ADU-S100 (+IP DMSO diluted in PBS) or their combination. Mice were treated under isoflurane anesthesia. Tumors were measured every 3–4 days using electronic calipers, and tumor volumes were calculated by using the formula (*L* × *W* × *W*)/2.

### Tumor digestion and flow cytometry

At the indicated times, mice were sacrificed, cardiac perfusion was performed and tumors were extracted. A small tumor chunk was snap-frozen for RNA analysis and the remainder of the tumor was minced, blended with the gentleMACS Dissociator (Miltenyi Biotec), and digested with the MACS Miltenyi Tumor Dissociation Kit (Miltenyi Biotec #130-096-730). Dissociated tumor cells were washed with RPMI-1640 medium and lysed with RBC Lysis Solution (Qiagen). Cells were resuspended in FACS buffer: PBS (Life Technologies) containing 0.5% BSA and 2 mmol/L EDTA (Sigma-Aldrich). The Zombie Aqua Fixable Viability Kit was applied to cells in combination with anti-mouse CD16/CD32 Fcγ receptor II/III blocking antibody (Affymetrix #14-0161-85) for 20 min at room temperature, prior to incubation with primary antibodies for 1 h at 4 °C. Cells were fixed and permeabilized using the FOXP3/Transcription Factor Staining Buffer Set (Affymetrix #00-5523-00) and incubated with antibodies for intracellular antigens overnight at 4 °C. Cells were subsequently washed, resuspended in PBS, and analyzed using a BD LSRFortessa flow cytometer. Compensation was performed manually on BD FACSDiva using single color and isotype controls. Signal threshold definition was defined using all-stain, unstained, and isotype controls and analysis was performed on FlowJo V10. Gating strategies are shown in Supplementary Fig. 4. The following fluorophore-conjugated primary antibodies were used in flow cytometry studies: Alexa Fluor^®^ 488 anti-mouse CD45 (1:500) (BioLegend #103122), Alexa Fluor^®^ 594 anti-mouse CD3 (1:1000) (BioLegend #100240), PE/Cyanine7 anti-mouse CD8a (1:1000) (BioLegend #100721), PE anti-mouse CD4 (1:1000) (BioLegend #100408), Alexa Fluor^®^ 647 anti-human/mouse Granzyme B (1:150) (BioLegend #515405), Alexa Fluor^®^ 647 mouse IgG1κ Isotype Ctrl (1:750) (BioLegend #400135), mouse FoxP3 PerCP/Cy5.5 (1:100) (BD Biosciences #563902), PerCP/Cy5.5 Rat IgG2aκ Isotype Ctrl Antibody (1:100) (BioLegend #400531), Brilliant Violet 605™ anti-T-bet (1:50) (BioLegend #644817), Brilliant Violet 711™ anti-mouse/human CD11b (1:1000) (BioLegend #101241), Brilliant Violet 650™ anti-mouse CD11c (1:500) (BD Biosciences #564079), FITC anti-mouse CD40 (1:125) (BioLegend #124607), Brilliant Violet 421™ anti-mouse I-A/I-E (MHCII) (1:1000) (BioLegend #107631).

### nanoString immune gene expression analysis

RNA isolation from snap-frozen tumor chunks was performed at the Center for Advanced Molecular Diagnostics (CAMD; Brigham and Women’s Hospital) using the Maxwell RSC Tissue kit. RNA was quantified by Nanodrop Spectrophotometer. RNA (100 ng) was loaded into the nCounter^®^ PanCancer Immune Profiling Panel, consisting of 770 genes, on the nanoString instrument. Data were analyzed using the Advanced Analysis Module of the nSolver^TM^ analysis software 4.0 (NanoString Technologies) and subjected to Quality control, and Background correction and Normalization against positive controls and housekeeping genes. The geometric mean of eight housekeeping genes was used to calculate normalization factors. Raw counts below the negative controls were discarded from further analysis.

### Statistical analyses

Statistical analyses were performed using GraphPad Prism v8. For comparison of 2 sets of measurements, unpaired *t*-test was performed. Unpaired *t*-test with Welch correction was used when sample variances were not equal, as defined by the Brown–Forsythe test. For comparison of 3 or more sets of unpaired measurements, one-way ANOVA was performed with Holm-Sidak’s multiple comparisons test on pre-selected relevant pairs. *P* values are indicated on the graphs.

### Reporting summary

Further information on research design is available in the Nature Research Reporting Summary linked to this article.

## Supplementary information

Supplementary Information

Reporting Summary

## Data Availability

Nanostring data described in this study are deposited in NCBI’s Gene Expression Omnibus (GEO) database under accession number GSE204858. Additional relevant data that support the findings of this study are available from the authors upon reasonable request.
