# Supplementary material for: STING agonism enhances anti-tumor immune responses and therapeutic efficacy of PARP inhibition in BRCA-associated breast cancer
Source: NPJ Breast Cancer. 2022 Sep 6;8:102. doi: 10.1038/s41523-022-00471-5 (PMC9448789; doi:10.1038/s41523-022-00471-5)

Supplementary Figures

Supplementary Figure 1

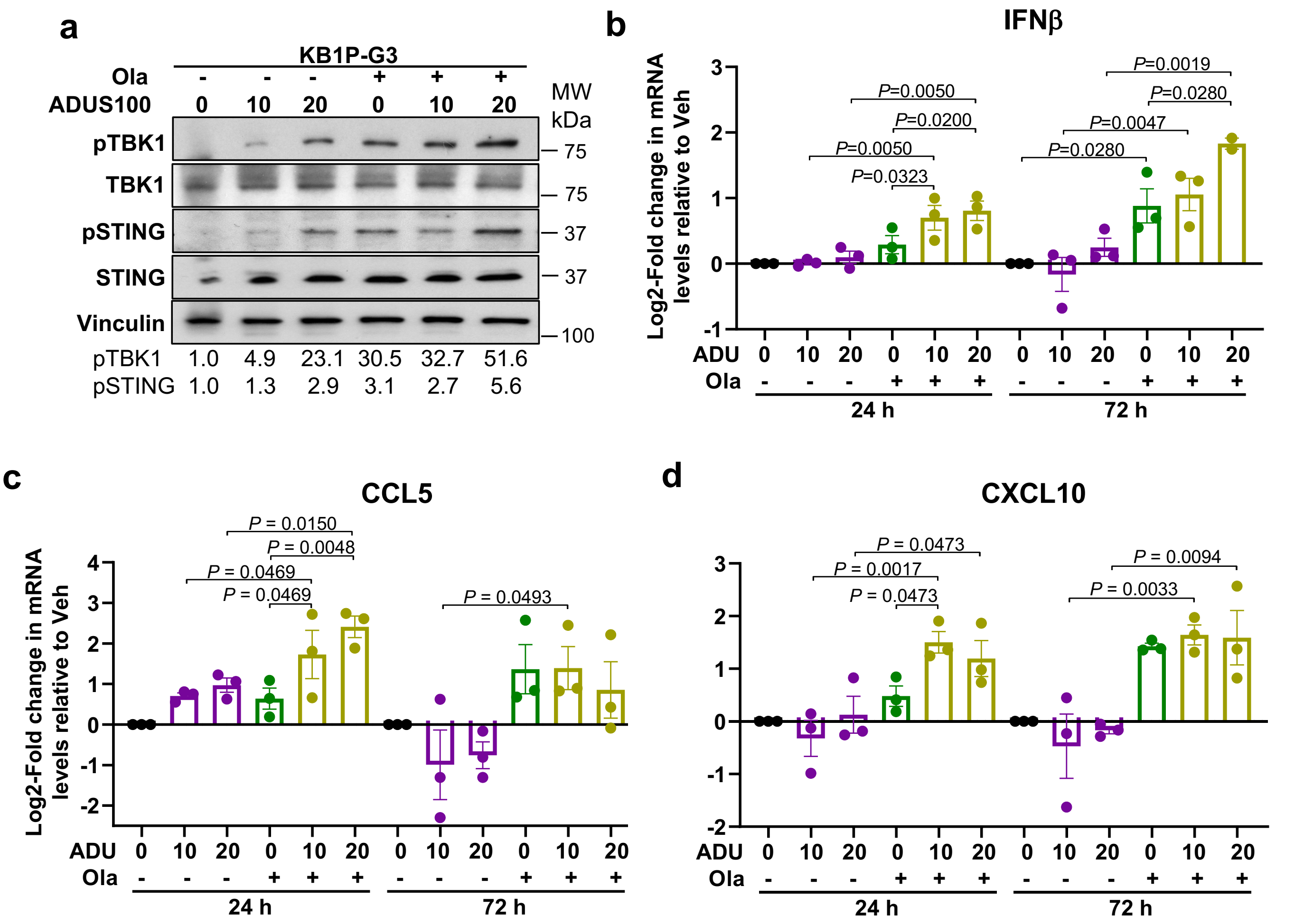

Supplementary Fig. 1. STING agonism enhances STING pathway activation and proinflammatory cytokine production in response to PARP inhibition in murine TNBC cells. The HR-deficient TNBC cell line KB1P-G3 (murine; derived from the K14-*CreBRCA1*$^{f/f}$*TP53*$^{f/f}$ GEMM) was treated with vehicle (-, 0), 1 µM olaparib, the indicated doses of ADU-S100 (µg/ml) or their combination. **(a)** At 72 hours post-treatment, protein was extracted for immunoblot analysis of phospho-TBK1 (Ser172), total TBK1, phospho-STING (Ser366 in human and Ser365 in murine) and total STING expression with vinculin as loading control. Numbers below the blots represent phosphorylated protein levels quantified by densitometric analysis. Immunoblots are representative of 3 independent experiments. **(b-d)** At 24 and 72 hours, post-treatment RNA was extracted and used for qPCR analysis of **(b)** IFNβ, **(c)** CCL5 and **(d)** CXCL10 mRNA expression, plotted as log2-fold change versus vehicle. Statistical analyses were performed using one-way ANOVA with Holm-Sidak post hoc test. Error bars represent standard error of the mean (S.E.M.) of 3 independent experiments. In murine cells, the combination resulted in greater IFNβ, CCL5 and CXCL10 mRNA expression at 24 hours compared to monotherapies, with effects more comparable to olaparib alone at 72 hours.

Supplementary Figure 2

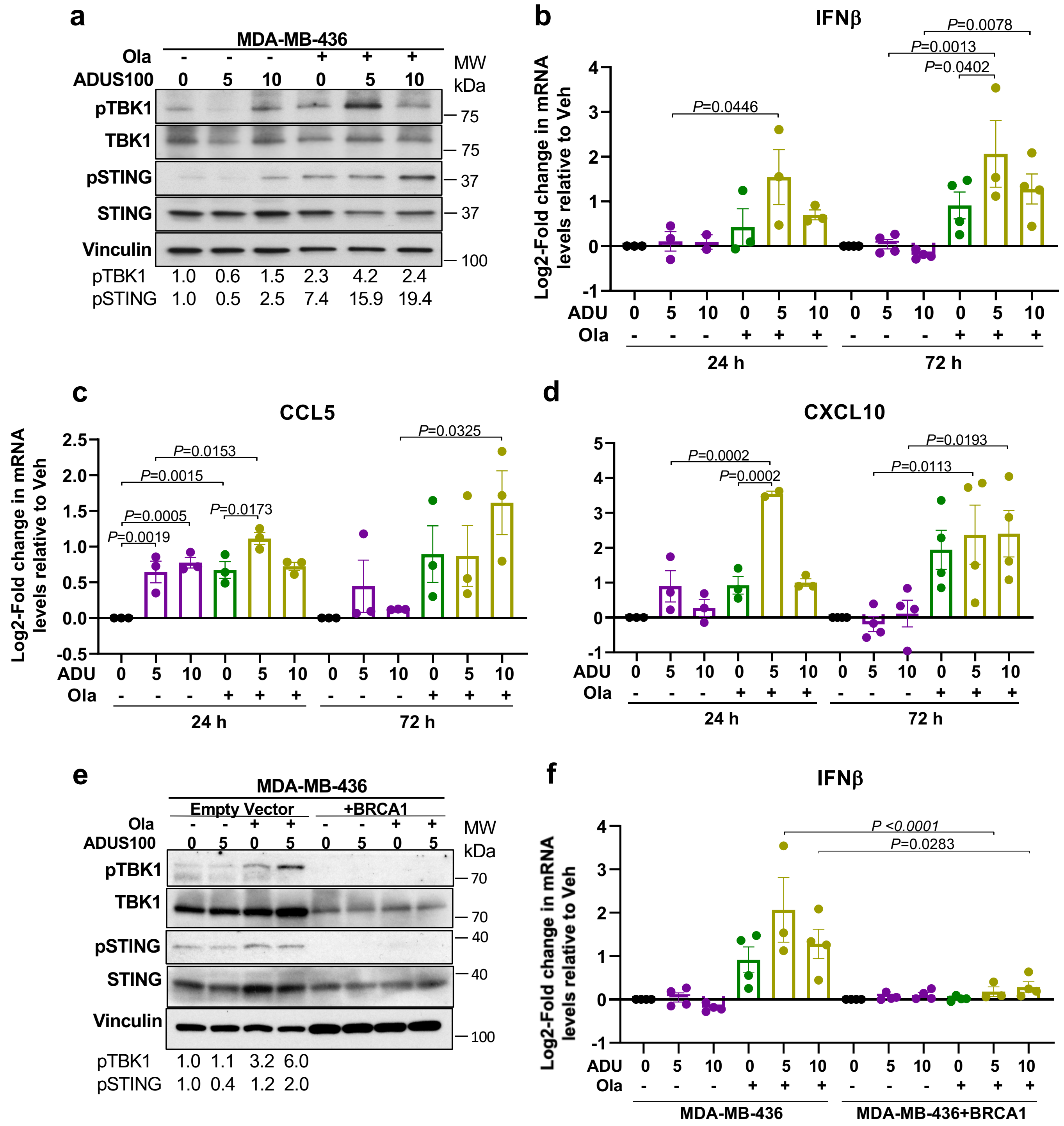

Supplementary Fig. 2. STING agonism enhances STING pathway activation and proinflammatory cytokine production in response to PARP inhibition in human *BRCA1*-mutant TNBC cells. The HR-deficient *BRCA1*-mutant TNBC cell line MDA-MB-436 (Johnson et al., *Proc Natl Acad Sci USA*, 2013; Johnson et al., *Cell Rep*, 2016) was treated with vehicle (-, 0), 1 μM olaparib, the indicated doses of ADU-S100 (μg/ml) or their combination. **(a)** At 72 hours post-treatment protein was extracted for immunoblot analysis of phospho-TBK1 (Ser172), total TBK1, phospho-STING (Ser366 in human and Ser365 in murine) and total STING expression with vinculin as loading control. Numbers below the blots represent phosphorylated protein levels quantified by densitometric analysis. Immunoblots are representative of 3 independent experiments. **(b-d)** At 24 and 72 hours, post-treatment RNA was extracted and used for qPCR analysis of **(b)** IFNβ, **(c)** CCL5 and **(d)** CXCL10 mRNA expression, plotted as log2-fold change versus vehicle. Statistical analyses were performed using one-way ANOVA with Holm-Sidak post hoc test. Error bars represent standard error of the mean (S.E.M.) of 3 independent experiments. Similar patterns emerged in human cells compared to murine cells, although the lower dose of ADU-S100 combined with PARP inhibition resulted in more productive inflammatory responses, consistent with a previous report that higher doses of ADU-S100 can compromise tumor-specific T-cell responses and durable anti-tumor immunity (Sivick et al. *Cell Rep*, 2018). Of note, at certain doses and time points, ADU-S100 monotherapy decreased proinflammatory cytokine production, suggesting the presence of inhibitory feedback loops, as has previously been described after STING pathway activation (Pantelidou et al. *Cancer Discov*, 2019; Flood et al. *Immunol Rev*, 2019).

Supplementary Figure 3

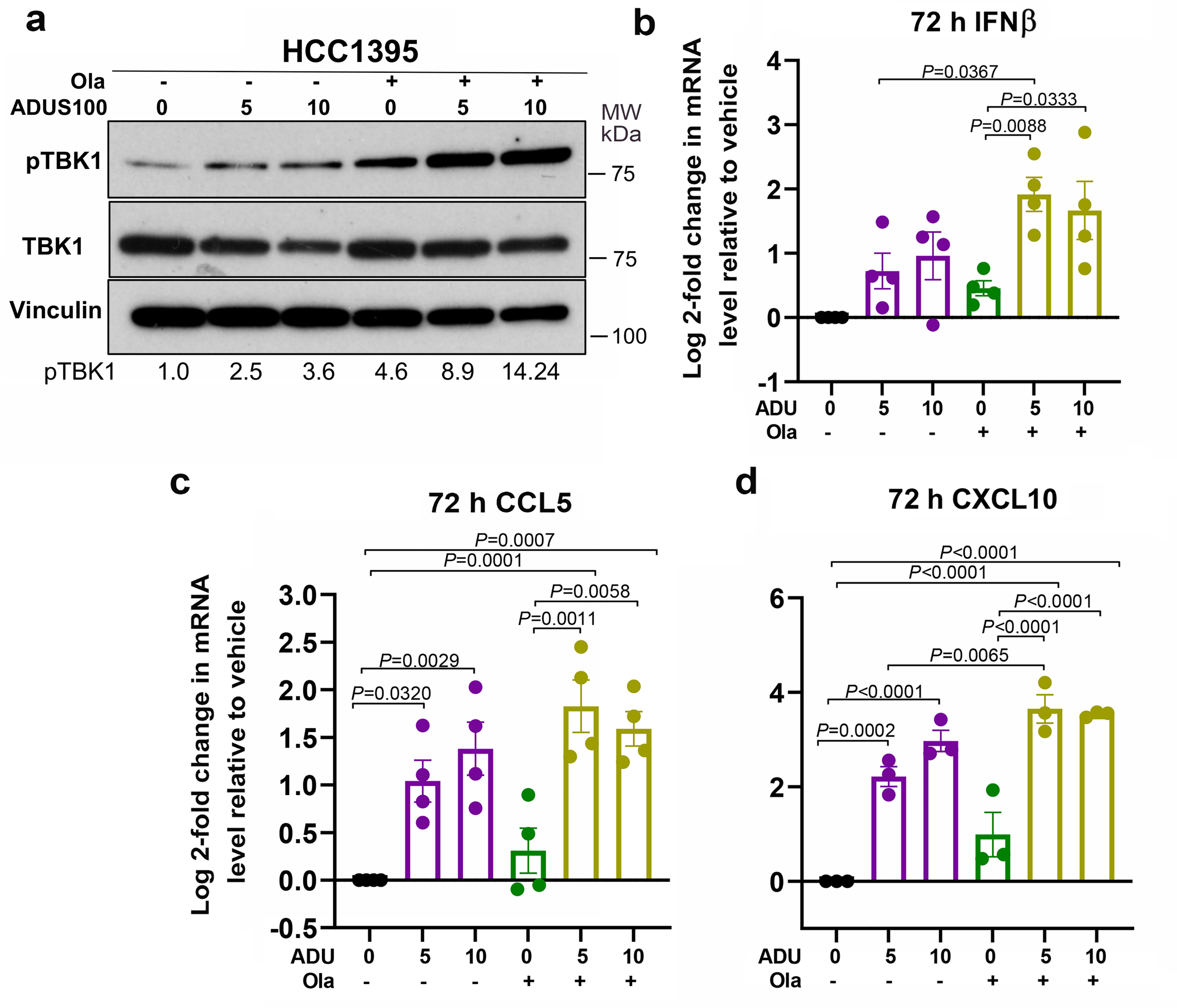

Supplementary Fig. 3. STING agonism enhances STING pathway activation and proinflammatory cytokine production in response to PARP inhibition in human *BRCA2*-mutant TNBC cells. The HR-deficient *BRCA1/BRCA2*-mutant TNBC cell line HCC1395 (Lehmann et al., *J Clin Invest*, 2011; Johnson et al., *Cell Rep*, 2016) was treated with vehicle (-, 0), 1 µM olaparib, the indicated doses of ADU-S100 (µg/ml) or their combination. **(a)** At 72 hours post-treatment protein was extracted for immunoblot analysis of phospho-TBK1 (Ser172) and total TBK1, with vinculin as loading control. Numbers below the blots represent phosphorylated protein levels quantified by densitometric analysis. Immunoblots are representative of 3 independent experiments. **(b-d)** At 72 hours, post-treatment RNA was extracted and used for qPCR analysis of **(b)** IFNβ, **(c)** CCL5 and **(d)** CXCL10 mRNA expression, plotted as log2-fold change versus vehicle. Statistical analyses were performed using one-way ANOVA with Holm-Sidak post hoc test. Error bars represent standard error of the mean (S.E.M.) of 3 independent experiments. Trends with combination treatment were similar to those observed in KB1P-G3 and MDA-MB-436 cells.

Supplementary Figure 4

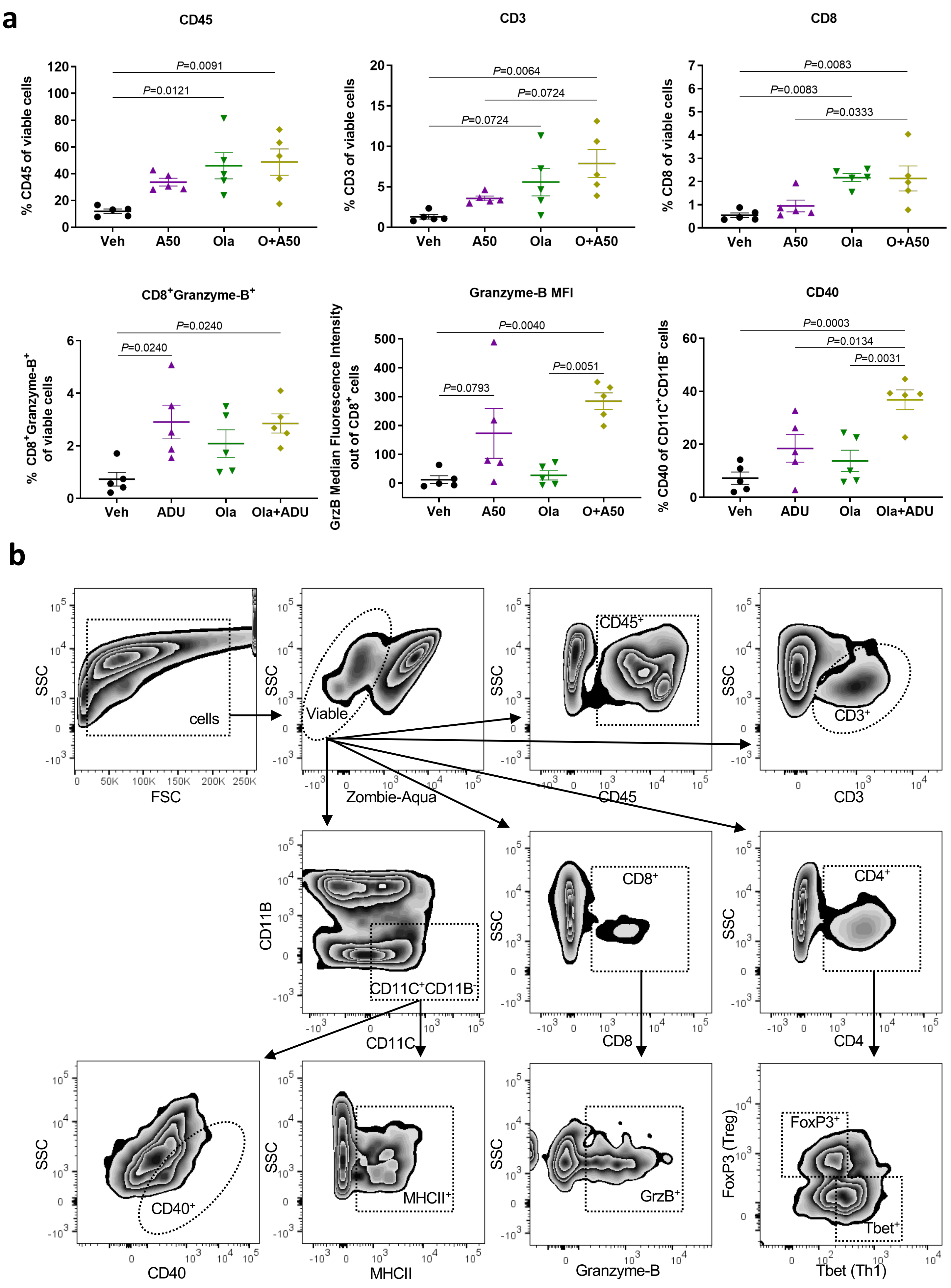

Supplementary Fig. 4. Immunologic effects of combined PARP inhibition and STING agonism in a BRCA-deficient GEMM at 7 days. (a) Tumor chunks from the K14-*Cre-Brca1*$^{f/f}$*Tp53*$^{f/f}$ GEMM were transplanted in syngeneic FVB/129P mice, which were treated with vehicle, olaparib, ADU-S100 or their combination (4-5 mice/group). At 7 days tumors were harvested, and single-cell suspensions were subjected to flow cytometry. Scatter plots show CD45$^+$ cells, CD3$^+$ cells, CD8$^+$ and CD4$^+$ T-cells, granzyme-B$^+$ CD8 T-cells, granzyme-B median fluorescence intensity (MFI) in CD8 cells, and CD40$^+$ CD11C$^+$CD11B$^-$ dendritic cells. Statistical analyses were performed using one-way ANOVA with Holm-Sidak post hoc test. Error bars are S.E.M. **(b)** Gating strategy used in the flow cytometric analysis of immune cell subsets. Debris was excluded on SSC vs FSC plot and zombie aqua-negative, i.e., viable cells were gated. Live cells were analyzed for expression of CD45 (hematopoietic cells), CD3 (total T cells), CD8 T-cells, CD4 T-cells and dual expression of CD11B/CD11C. CD8$^+$ T-cells were further analyzed for expression of Granzyme-B and median fluorescence intensity (MFI) was derived. CD4 T-cells were analyzed for expression of Tbet and FoxP3. CD11C$^+$CD11B$^-$ dendritic cells were analyzed for expression of CD40 and MHCII.

Supplementary Figure 5

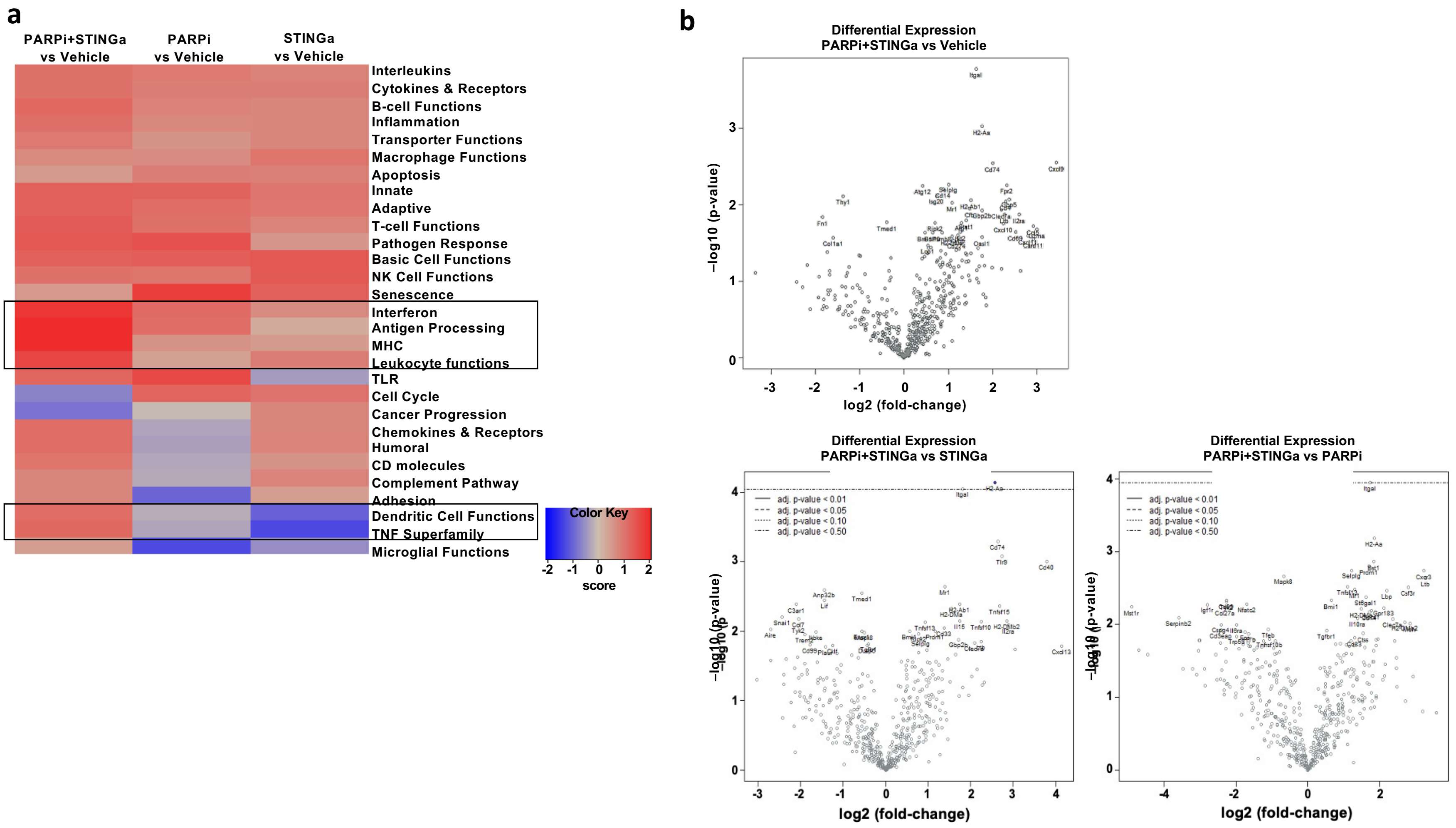

Supplementary Fig. 5. The combination of STING agonism with PARP inhibition upregulates the expression of genes involved in antigen processing, interferon responses and leukocyte functions. *K14-Cre-Brca*$^{f/f}$*Tp53*$^{f/f}$ tumors from mice treated as in Figure 2 with vehicle, olaparib, ADU-S100 or their combination were harvested at 3 days. RNA was isolated from 3 tumors per group and subjected to nanoString mRNA expression analysis using the nCounter® PanCancer Immune Profiling Panel. **(a)** NanoString Gene Set Analysis (GSA) heatmap of directed global significance scores. The scores measure the extent to which a gene set is up- or down-regulated relative to the covariate and they are calculated as the square root of the mean signed squared *t*-statistic for the genes in a gene set, with *t*-statistics coming from the linear regression underlying differential expression analysis. Red denotes gene sets whose genes exhibit extensive over-expression with the covariate, blue denotes gene sets with extensive under-expression. Boxes highlight the most upregulated pathways in response to the combination of PARPi and STINGa. **(b)** Differential expression volcano plots displaying each target's -log10 (p-value) and log2 fold change in response to the PARPi/STINGa combination treatment versus vehicle treatment or STINGa or PARPi as single treatments. Highly statistically significant targets fall at the top of the plot, and highly differentially expressed genes fall to either side. *P* values were generated using unpaired *t*-tests with Benjamini-Yekutieli false discovery rate (FDR) adjustment.

Supplementary Figure 6

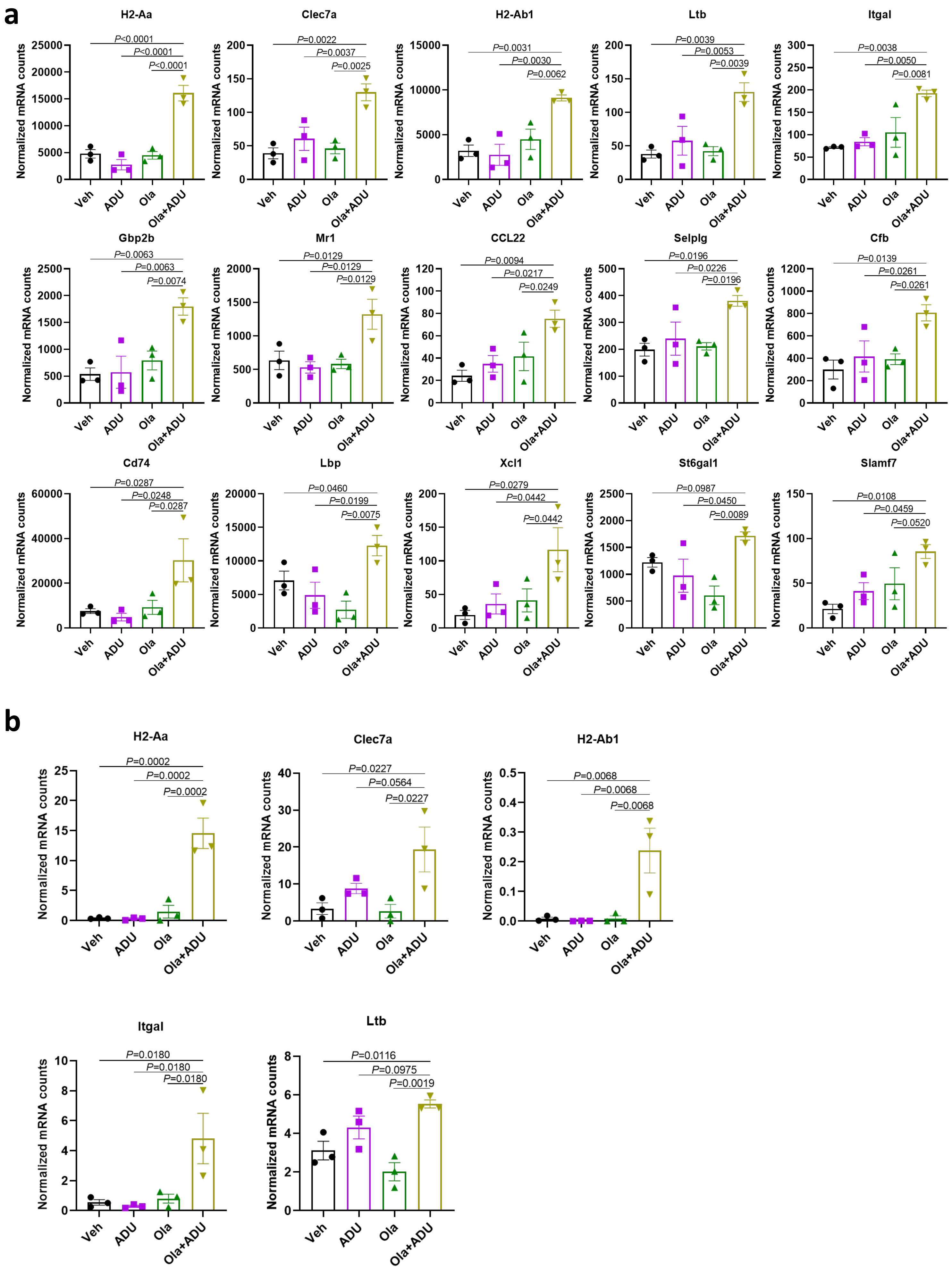

Supplementary Fig. 6. Top upregulated genes from nanoString analysis and validation by qPCR. (a) Top 15 most significantly upregulated genes. Scatter plots demonstrate statistically significant increases in normalized RNA counts of indicated genes after treatment with PARPi+STINGa combination in comparison to single treatments. Statistical analyses were performed using one-way ANOVA with Holm-Sidak post hoc test. Error bars show S.E.M. **(b)** Scatter plots show significant increases in the RNA levels of H2-Aa, Clec7a, H2-Ab1, Itgal and Ltb genes (top 5 in the nanoString mRNA analysis) in response to the combination treatment as measured by qPCR.

Supplementary Figure 7

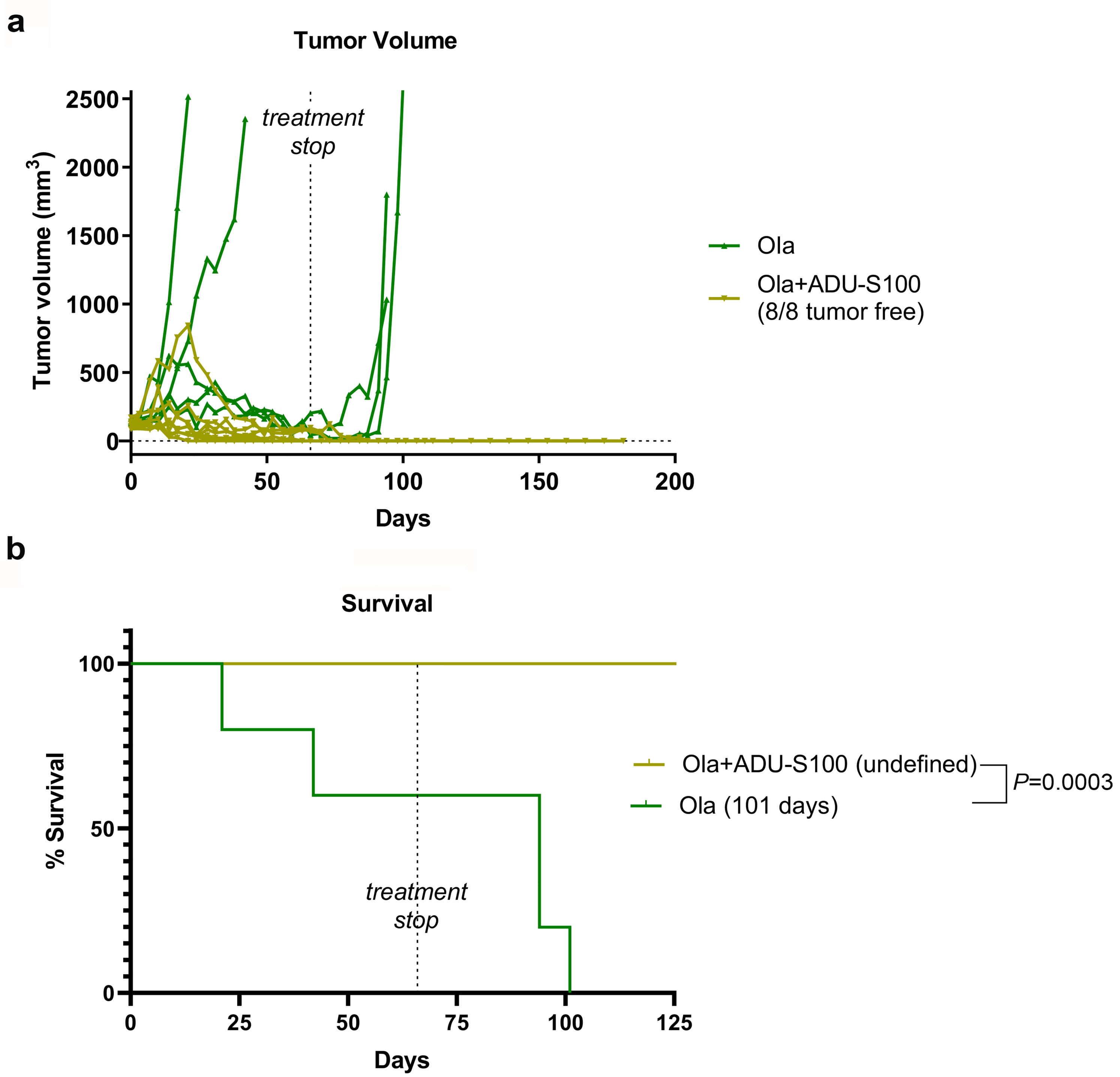

Supplementary Fig. 7. Efficacy of combined PARP inhibition and STING agonism *in vivo*. A second experiment was performed in which tumor chunks from the K14-Cre-Brca1f/fTp53f/f GEMM were transplanted in syngeneic FVB/129P mice, which were treated with olaparib +intratumoral saline or olaparib + ADU-S100 (5-8 mice/group). Tumor volume was measured twice per week and survival was recorded. **(a)** Tumor volumes in individual mice over time. **(b)** Percent survival. Median survival shown in brackets. Statistical analysis was performed using the Log-rank (Mantel-Cox) test (*P* values indicated). Intratumoral ADU-S100 injections were stopped when the tumor was cleared.

Supplementary Figure 8

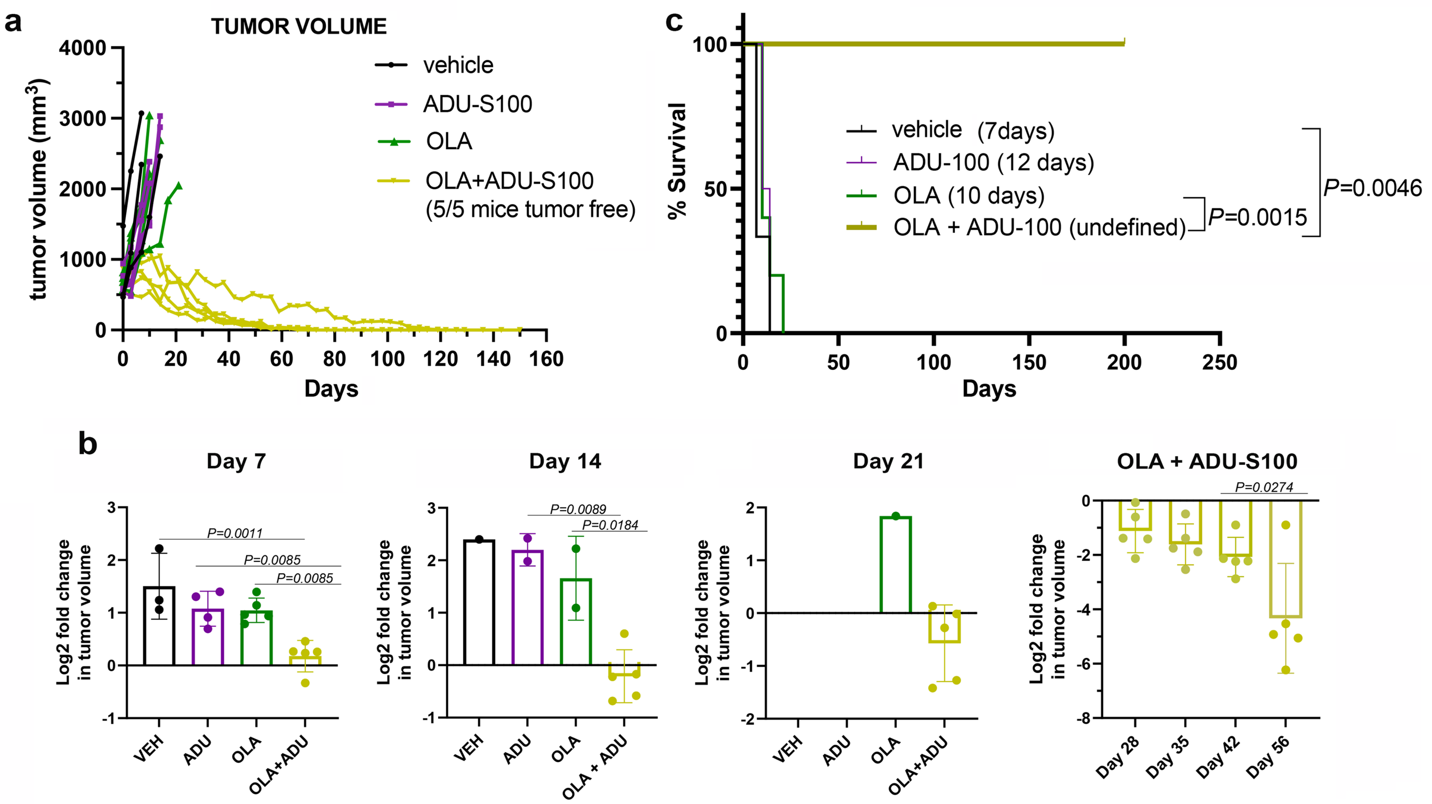

Supplementary Fig. 8. Efficacy of combined olaparib and ADU-S100 against established tumors with volumes ranging 500-1,000 mm^3. Tumor chunks from the *K14-Cre-Brca1$^{f/f}$Tp53$^{f/f}$* GEMM were transplanted in syngeneic FVB/129P mice, which were treated with vehicle, olaparib (daily), ADU-S100 (weekly) or their combination (3-5 mice/group) after reaching a volume ranging 500-1,000 mm^3. Tumor volume was measured twice weekly and survival recorded. **(a)** Tumor volumes in individual mice over time. **(b)** Log2 fold-change in tumor volumes at the indicated time points and treatments. Statistical analyses were performed using one-way ANOVA with Holm-Sidak post hoc test. **(c)** Percent survival. Median survival shown in brackets. Statistical analysis was performed using the Log-rank (Mantel-Cox) test. Intratumoral ADU-S100 injections were stopped when the tumor was cleared.

Supplementary Figure 9

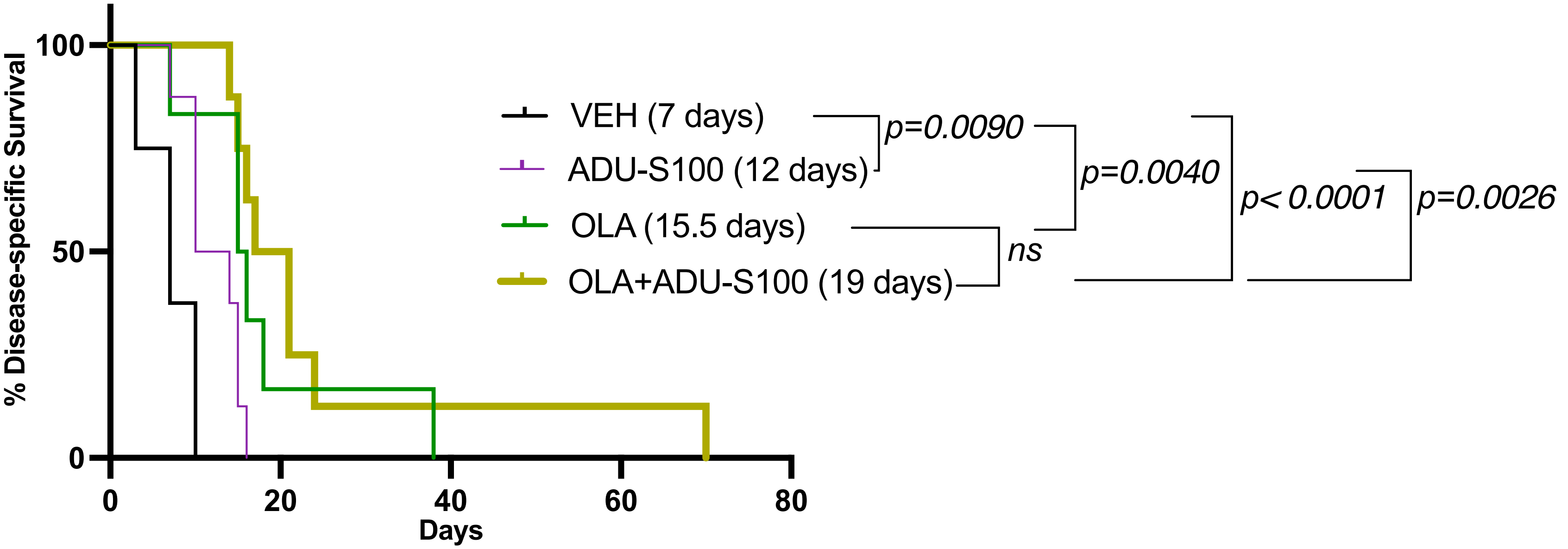

Supplementary Fig. 9. An intact immune system is required for the optimal efficacy of combined PARP inhibition and STING agonism. Tumor chunks from the *K14-Cre-Brca1$^{f/f}$Tp53$^{f/f}$* GEMM were transplanted in immunodeficient NSG mice, which were treated with vehicle, olaparib (daily), ADU-S100 (weekly) or their combination (6-8 mice/group). Tumor volume was measured twice weekly and survival recorded. Percent survival is graphed with median survival shown in brackets. Statistical analysis was performed using the Log-rank (Mantel-Cox) test.

SUPPLEMENTARY REFERENCES

Flood, B. A., Higgs, E. F., Li, S., Luke, J. J. & Gajewski, T. F. STING pathway agonism as a cancer therapeutic. *Immunol Rev* **290**, 24-38 (2019).

Johnson N., *et al.* Stabilization of mutant BRCA1 protein confers PARP inhibitor and platinum resistance. *Proc Natl Acad Sci USA* **110**, 17041-17046 (2013).

Johnson S.F., *et al*. CDK12 inhibition reverses de novo and acquired PARP inhibitor resistance in *BRCA* wild-type and mutated models of triple-negative breast cancer. *Cell Rep* **17**, 2367-2381 (2016).

Lehmann B.D., *et al*. Identification of human triple-negative breast cancer subtypes and preclinical models for selection of targeted therapies. *J Clin Invest* **121**, 2750-2767 (2011).

Pantelidou, C. *et al.* PARP Inhibitor Efficacy Depends on CD8(+) T-cell Recruitment via Intratumoral STING Pathway Activation in BRCA-Deficient Models of Triple-Negative Breast Cancer. *Cancer Discov* **9**, 722-737 (2019).

Sivick, K. E. *et al.* Magnitude of Therapeutic STING Activation Determines CD8(+) T Cell-Mediated Anti-tumor Immunity. *Cell Rep* **25**, 3074-3085 (2018).

<u>Supplementary Table 1</u>: Directed Global Significance Scores - differential expression in each treatment group vs baseline of vehicle

Gene Set	Olaparib+ADU-S100	Olaparib	ADU-S100
Antigen Processing	2.084	1.14	0.295
MHC	2.075	0.625	0.512
Interferon	1.922	1.18	0.748
Leukocyte Functions	1.69	0.442	0.939
Pathogen Response	1.457	1.54	0.602
T-Cell Functions	1.414	1.095	0.834
Innate	1.337	1.296	1.034
Basic Cell Functions	1.325	1.355	1.457
Adaptive	1.32	1.145	1.02
TLR	1.269	1.656	-0.393
B-Cell Functions	1.231	0.874	0.807
TNF Superfamily	1.212	-0.288	-1.291
Humoral	1.169	-0.352	0.842
Chemokines & Receptors	1.166	-0.295	0.796
Dendritic Cell Functions	1.156	-0.202	-1.015
Inflammation	1.137	0.768	0.805
Cytokines & Receptors	1.106	0.903	0.904
Interleukins	1.106	0.981	0.854
NK Cell Functions	1.081	1.044	1.445
CD molecules	1.044	-0.277	0.631
Transporter Functions	0.97	0.626	0.786
Complement Pathway	0.808	-0.256	0.822
Adhesion	0.801	-0.984	0.478
Macrophage Functions	0.752	0.734	1.042
Senescence	0.552	1.8	1.324
Apoptosis	0.535	0.916	0.883
Microglial Functions	0.485	-1.278	-0.546
Cell Cycle	-0.659	1.275	1.073
Cancer Progression	-0.815	-0.072	0.804

<u>Supplementary Table 2</u>: Annotation of top upregulated genes identified by nanoString analysis in response to STINGa+PARPi treatment

nCounter® Mouse PanCancer Immune Profiling Panel - Annotations			
#	Gene Name	Protein	Annotation
1	***H2-Aa***	H-2 class II histocompatibility antigen, A-B alpha chain	Antigen Processing, Interferon Response, Mature T-Cell Functions, MHC class I & II
2	***Clec7a***	C-type lectin domain family 7 member A	Inflammatory Response, Innate Response, Leukocyte Functions, Phagocytosis
3	***H2-Ab1***	H-2 class II histocompatibility antigen, A-K beta chain	Antigen Processing, Interferon Response, Mature T-Cell Functions, MHC class I & II
4	***Ltb***	Lymphotoxin-beta	Cytokines, Interleukins, TNF Superfamily Members
5	***Itgal***	Integrin alpha L	Adhesion, CD molecules, Leukocyte Functions, Mature T-Cell Functions
6	***Gbp2b***	Interferon-induced guanylate-binding protein 1	Basic Cell Functions
7	***Mr1***	Major histocompatibility complex class I-related gene protein	Antigen Processing, MHC class I & II
8	***Ccl22***	C-C motif chemokine 22	Chemokines, Cytokines, Humoral Response, Inflammatory Response, Regulation of Inflammatory Response
9	***Selplg***	P-selectin glycoprotein ligand 1	Adhesion, CD molecules, Leukocyte Functions
10	***Cfb***	Complement factor B	Complement Pathway, Innate Response
11	***Cd74***	Cluster of Differentiation 74 (HLA class II histocompatibility antigen gamma chain)	Antigen Processing, CD molecules, Innate Response, Mature B-Cell Functions, MHC class I & II, T-Cell Differentiation
12	***Lbp***	Lipopolysaccharide-binding protein	Chemokines, Inflammatory Response, Innate Response, Leukocyte Functions, Macrophage Functions, Transmembrane Transportation
13	***Xcl1***	Lymphotactin	Chemokines, Cytokines, Cytotoxicity, Inflammatory Response, Innate Response, Mature T-Cell Functions
14	***St6gal1***	Beta-galactoside alpha-2,6-sialyltransferase 1	Basic Cell Functions, Humoral Response
15	***Slamf7***	Signaling lymphocytic activation molecule (SLAM) family member 7	CD molecules, Innate Response, Natural Killer Cell Functions

Source Files

Figure 2

Fig. 2e pSTING and STING (Control) pSTING and STING (STING KO)

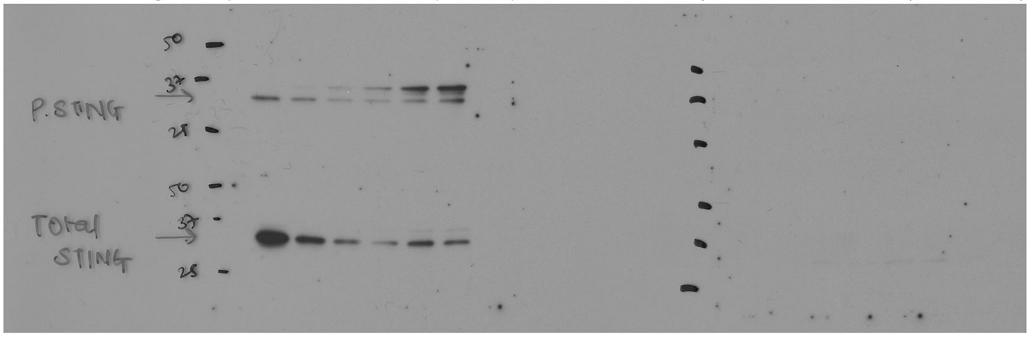

Fig. 2e vinculin blot (Control)

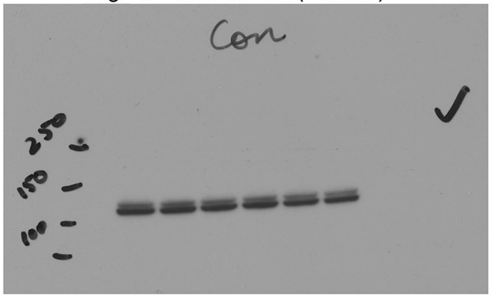

vinculin blot (STING KO)

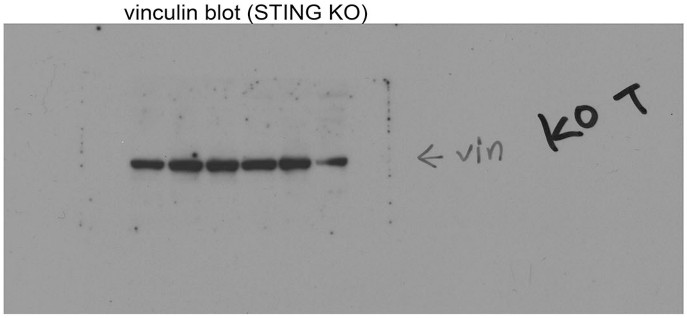

Fig. 2f STING (Control and KO)

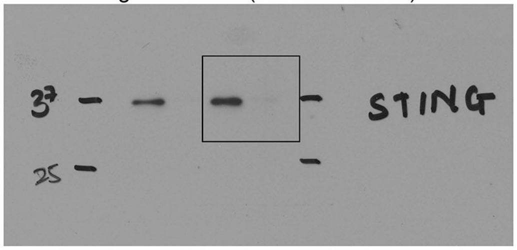

Tubulin (Control and KO)

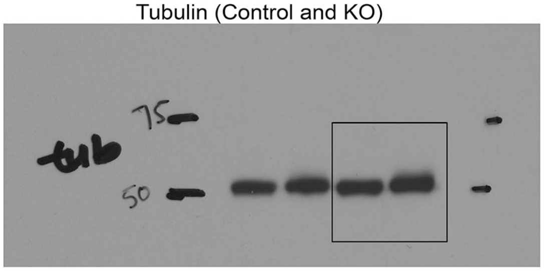

Supplementary Figure 1a

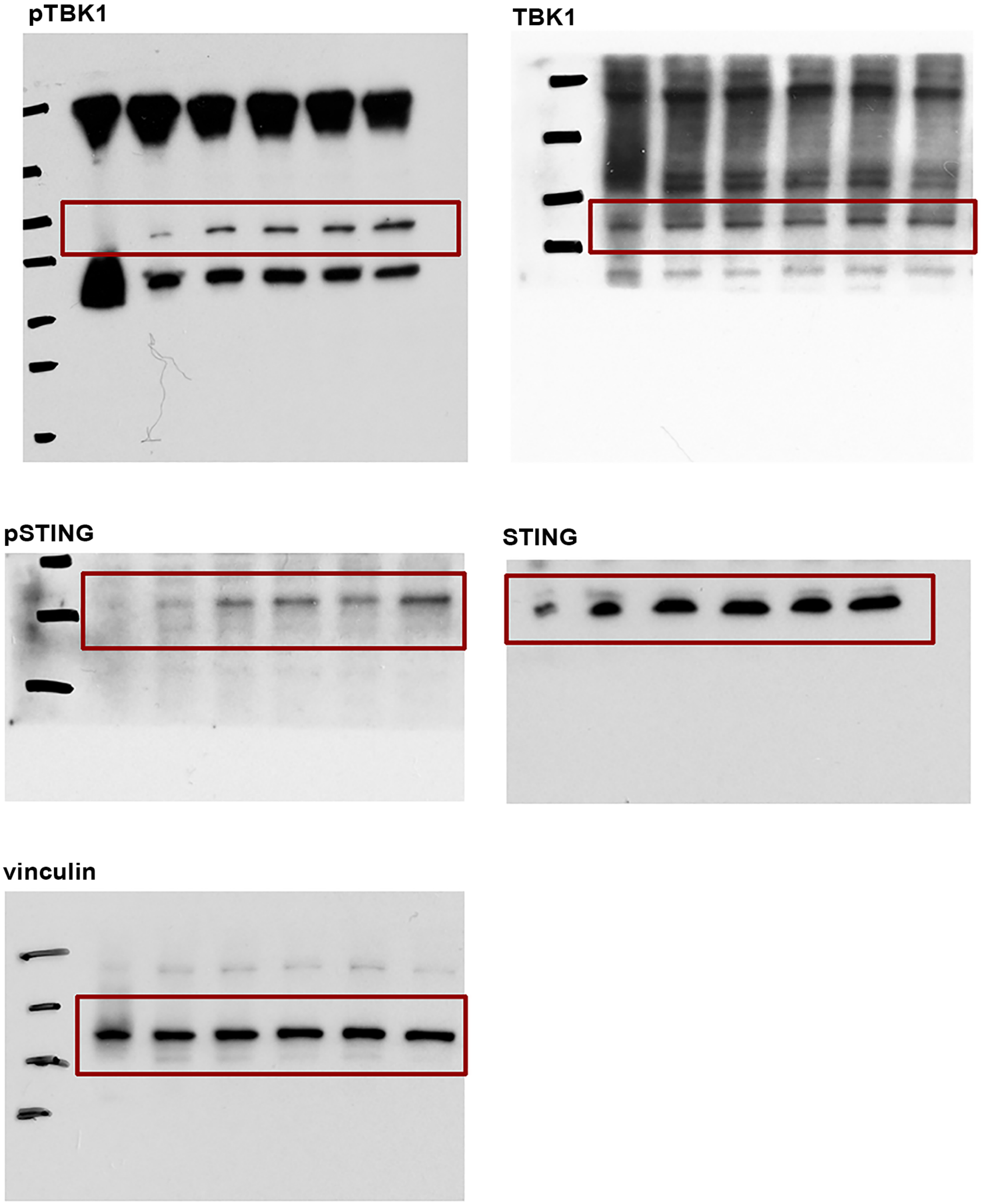

Supplementary Figure 2a

pTBK1

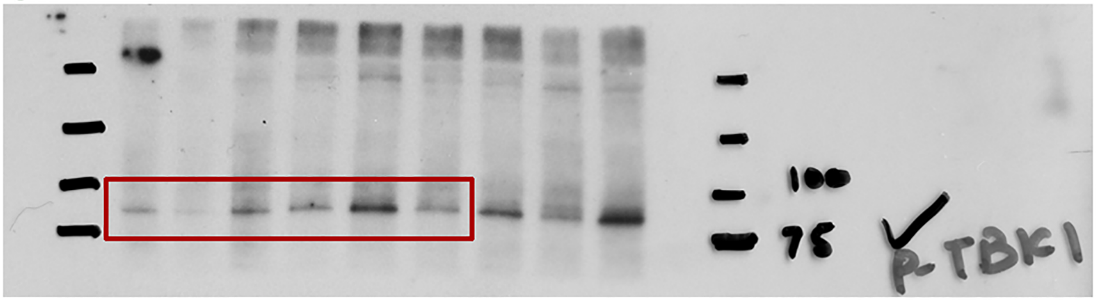

TBK1

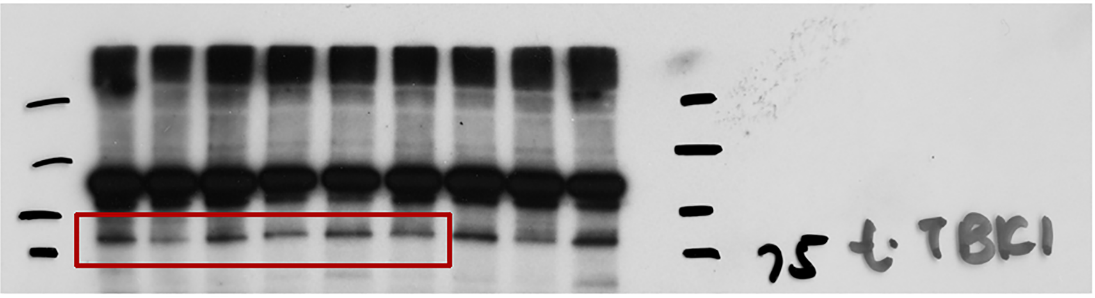

pSTING

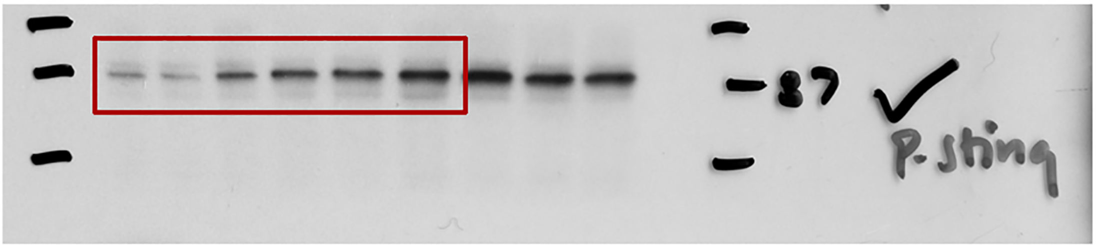

STING

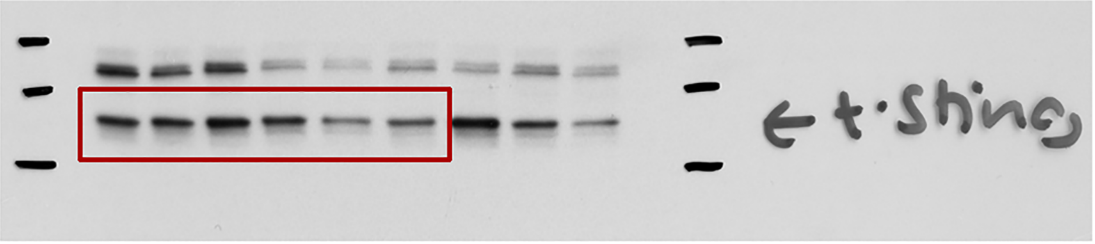

vinculin

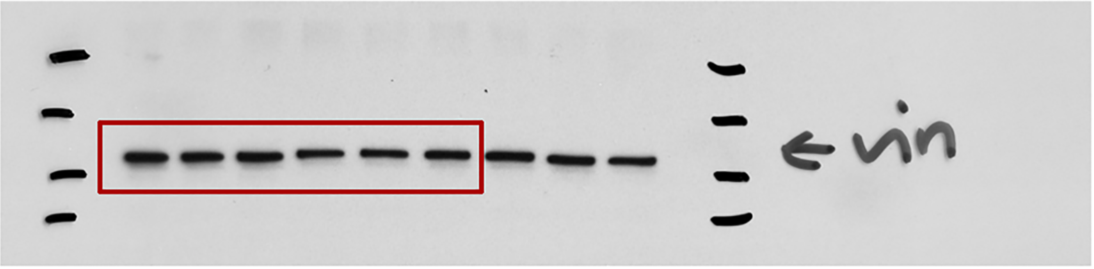

Supplementary Figure 2e

pTBK1

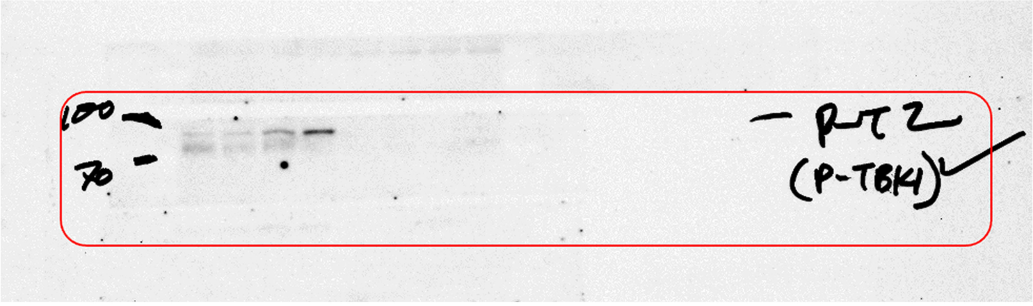

TBK1

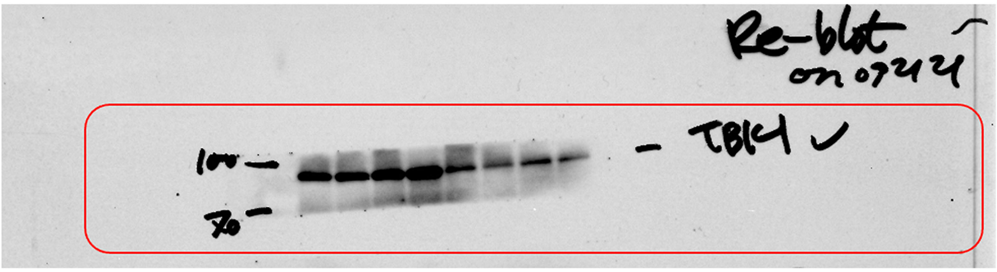

pSTING

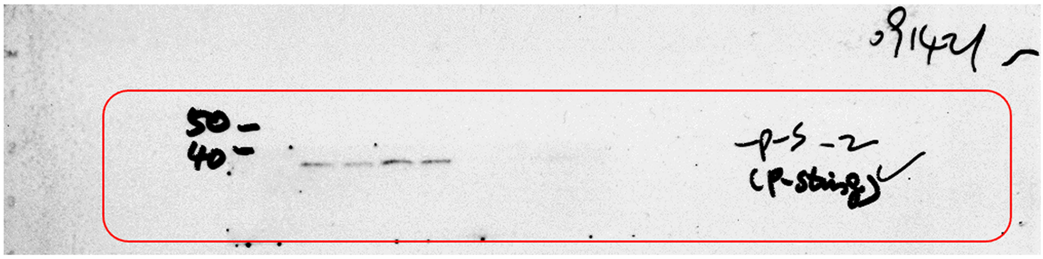

STING

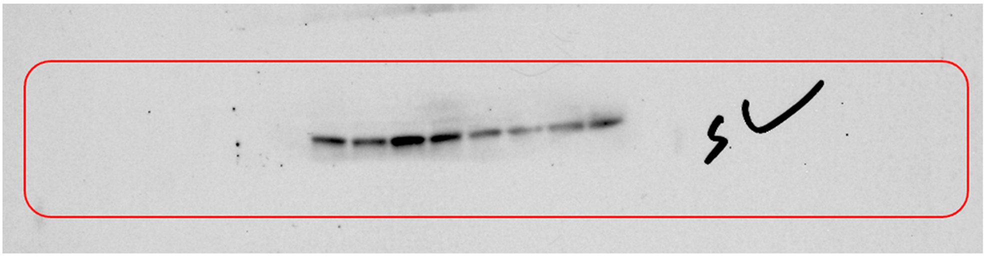

vinculin

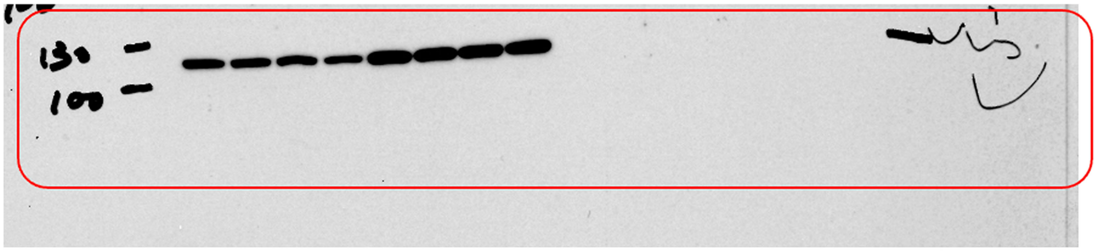

Supplementary Figure 3

pTBK1

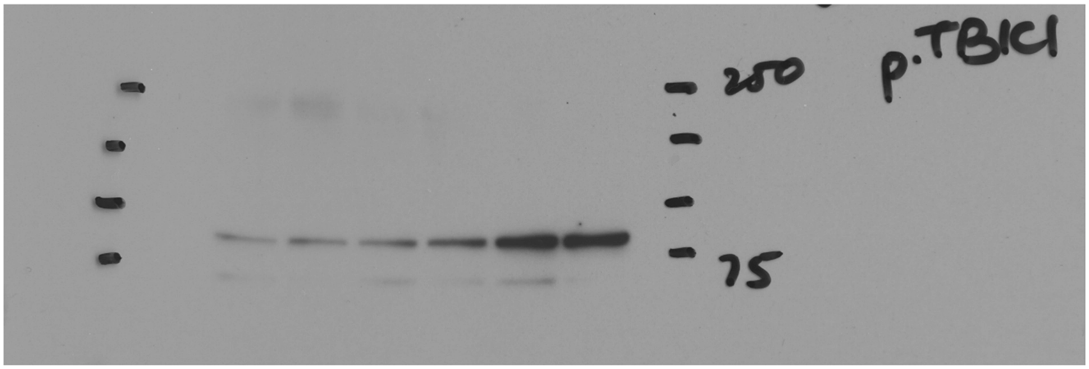

TBK1

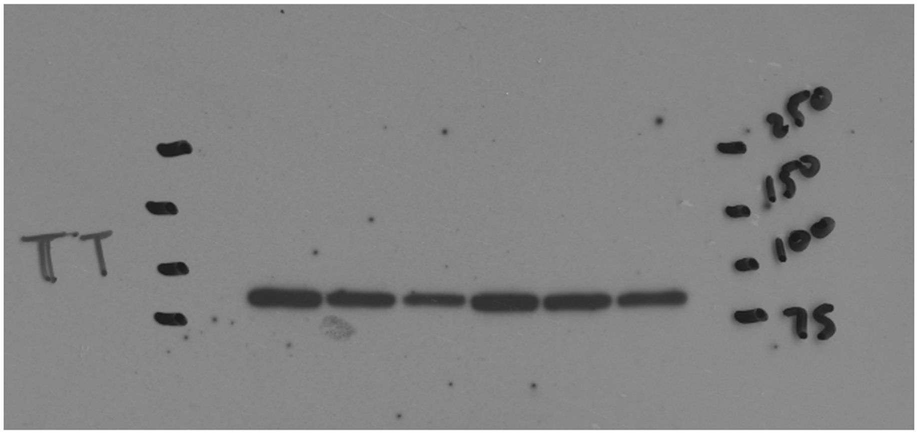

vinculin

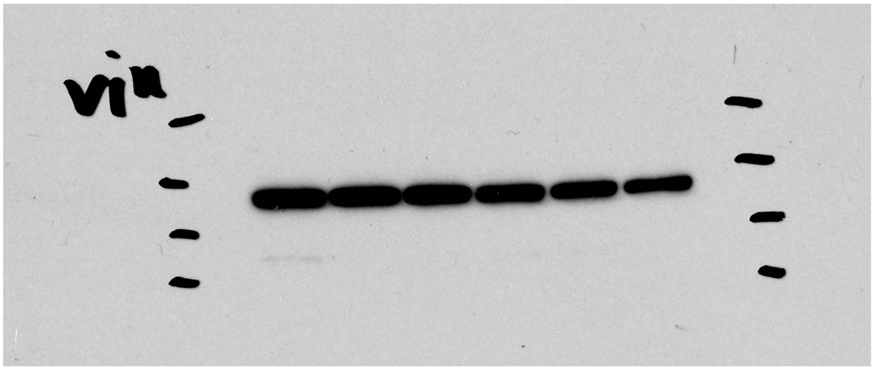

Supplement: Supplementary file 1 — Supplementary Information [file 41523_2022_471_MOESM1_ESM.pdf]
