# Supplementary material for: STING agonism enhances anti-tumor immune responses and therapeutic efficacy of PARP inhibition in BRCA-associated breast cancer
Source: NPJ Breast Cancer. 2022 Sep 6;8:102. doi: 10.1038/s41523-022-00471-5 (PMC9448789; doi:10.1038/s41523-022-00471-5)
Supplement: Supplementary file 2 — Reporting Summary [file 41523_2022_471_MOESM2_ESM.pdf]

nature portfolio

Corresponding author(s): Geoffrey I. Shapiro

Last updated by author(s): Jul 28, 2022

Reporting Summary

Nature Portfolio wishes to improve the reproducibility of the work that we publish. This form provides structure for consistency and transparency in reporting. For further information on Nature Portfolio policies, see our Editorial Policies and the Editorial Policy Checklist.

Statistics

For all statistical analyses, confirm that the following items are present in the figure legend, table legend, main text, or Methods section.

n/a	Confirmed	
☐	☒	The exact sample size (n) for each experimental group/condition, given as a discrete number and unit of measurement
☐	☒	A statement on whether measurements were taken from distinct samples or whether the same sample was measured repeatedly
☐	☒	The statistical test(s) used AND whether they are one- or two-sided<br>*Only common tests should be described solely by name; describe more complex techniques in the Methods section.*
☒	☐	A description of all covariates tested
☐	☒	A description of any assumptions or corrections, such as tests of normality and adjustment for multiple comparisons
☐	☒	A full description of the statistical parameters including central tendency (e.g. means) or other basic estimates (e.g. regression coefficient) AND variation (e.g. standard deviation) or associated estimates of uncertainty (e.g. confidence intervals)
☐	☒	For null hypothesis testing, the test statistic (e.g. F, t, r) with confidence intervals, effect sizes, degrees of freedom and P value noted<br>*Give P values as exact values whenever suitable.*
☒	☐	For Bayesian analysis, information on the choice of priors and Markov chain Monte Carlo settings
☒	☐	For hierarchical and complex designs, identification of the appropriate level for tests and full reporting of outcomes
☒	☐	Estimates of effect sizes (e.g. Cohen's d, Pearson's r), indicating how they were calculated

Our web collection on statistics for biologists contains articles on many of the points above.

Software and code

Policy information about availability of computer code

Data collection	nanoString nCounter® ; BD FACSDiva
Data analysis	Prism version 9; Image J version v1.53f51; nSolver analysis software 4.0 (NanoString Technologies); FlowJo V10;

For manuscripts utilizing custom algorithms or software that are central to the research but not yet described in published literature, software must be made available to editors and reviewers. We strongly encourage code deposition in a community repository (e.g. GitHub). See the Nature Portfolio guidelines for submitting code & software for further information.

Data

Policy information about availability of data

All manuscripts must include a data availability statement. This statement should provide the following information, where applicable:

- Accession codes, unique identifiers, or web links for publicly available datasets
- A description of any restrictions on data availability
- For clinical datasets or third party data, please ensure that the statement adheres to our policy

Nanostring data described in this study are deposited in NCBI’s Gene Expression Omnibus (GEO) database under accession number GSE204858 (https://www.ncbi.nlm.nih.gov/geo/query/acc.cgi?acc=GSE204858). Additional relevant data that support the findings of this study are available from the authors upon reasonable request.

Human research participants

Policy information about studies involving human research participants and Sex and Gender in Research.

Reporting on sex and gender	*Use the terms sex (biological attribute) and gender (shaped by social and cultural circumstances) carefully in order to avoid confusing both terms. Indicate if findings apply to only one sex or gender; describe whether sex and gender were considered in study design whether sex and/or gender was determined based on self-reporting or assigned and methods used. Provide in the source data disaggregated sex and gender data where this information has been collected, and consent has been obtained for sharing of individual-level data; provide overall numbers in this Reporting Summary. Please state if this information has not been collected. Report sex- and gender-based analyses where performed, justify reasons for lack of sex- and gender-based analysis.*
Population characteristics	*Describe the covariate-relevant population characteristics of the human research participants (e.g. age, genotypic information, past and current diagnosis and treatment categories). If you filled out the behavioural & social sciences study design questions and have nothing to add here, write "See above."*
Recruitment	*Describe how participants were recruited. Outline any potential self-selection bias or other biases that may be present and how these are likely to impact results.*
Ethics oversight	*Identify the organization(s) that approved the study protocol.*

Note that full information on the approval of the study protocol must also be provided in the manuscript.

Field-specific reporting

Please select the one below that is the best fit for your research. If you are not sure, read the appropriate sections before making your selection.

☒ Life sciences ☐ Behavioural & social sciences ☐ Ecological, evolutionary & environmental sciences

For a reference copy of the document with all sections, see nature.com/documents/nr-reporting-summary-flat.pdf

Life sciences study design

All studies must disclose on these points even when the disclosure is negative.

Sample size	Sample sizes (typically 6-8 mice/group) were chosen based on preliminary data on the effect of olaparib monotherapy in the K14Cre;BRCA1f/f;p53f/f genetically-engineered TNBC mouse model, in order to have adequate power (approximately 80%) to detect differences in tumor growth inhibition or tumor regression of 1.25 standardized units at 28 days. Fewer STING KO mice per group were adequate to demonstrate a statistically significant effect of olaparib/ADU-S100 treatment compared to olaparib alone (i.e., olaparib monotherapy is ineffective in the setting of intratumoral STING knockout).
Data exclusions	No data were excluded.
Replication	All attempts at replication were successful (e.g., Supplementary Fig. 7 confirmed the results shown in Fig. 2).
Randomization	Allocation of all mice to various treatment groups was randomized in each experiment.
Blinding	Flow cytometry and Nanostring analyses on tumors from treated mice were conducted in blinded fashion.

Reporting for specific materials, systems and methods

We require information from authors about some types of materials, experimental systems and methods used in many studies. Here, indicate whether each material, system or method listed is relevant to your study. If you are not sure if a list item applies to your research, read the appropriate section before selecting a response.

Materials & experimental systems

n/a	Involved in the study	
☐	☒	Antibodies
☐	☒	Eukaryotic cell lines
☒	☐	Palaeontology and archaeology
☐	☒	Animals and other organisms
☒	☐	Clinical data
☒	☐	Dual use research of concern

Methods

n/a	Involved in the study	
☒	☐	ChIP-seq
☐	☒	Flow cytometry
☒	☐	MRI-based neuroimaging

Antibodies

Antibodies used

The following primary antibodies were used: phospho-TBK1/NAK (Ser172) (D52C2) XP Rabbit mAb [Cell Signaling Technology (CST) #5483S] (for human cells), TBK1 (S172) (Abgent #AP7887a-ev) (for murine cells), TBK1/NAK (D1B4) Rabbit mAb (CST #3504S), phospho-STING (Ser366) (D7C3S) Rabbit mAb (CST #19781S) (for human cells), phospho-STING (Ser365) (D8F4W) Rabbit mAb (CST #72971S) (for murine cells), STING/TMEM173 (Novus Biologicals #NBP224683), Vinculin (CST #4650S).

The following fluorophore-conjugated primary antibodies were used in flow cytometry studies: Alexa Fluor® 488 anti-mouse CD45 (BioLegend #103122), Alexa Fluor® 594 anti-mouse CD3 (BioLegend #100240), PE/Cyanine7 anti-mouse CD8a (BioLegend #100721), PE anti-mouse CD4 (BioLegend #100408), Alexa Fluor® 647 anti-human/mouse Granzyme B (BioLegend #515405), Alexa Fluor® 647 mouse IgG1 κ Isotype Ctrl (BioLegend #400135), mouse FoxP3 PerCP/Cy5.5 (BD Biosciences #563902), PerCP/Cy5.5 Rat IgG2a, κ Isotype Ctrl Antibody (BioLegend #400531), Brilliant Violet 605™ anti-T-bet (BioLegend #644817), Brilliant Violet 711™ anti-mouse/human CD11b (BioLegend #101241), Brilliant Violet 650™ anti-mouse CD11c (BD Biosciences #564079), FITC anti-mouse CD40 (BioLegend #124607), Brilliant Violet 421™ anti-mouse I-A/I-E (MHCII) (BioLegend #107631).

Validation

Data detailing specificity and sensitivity of primary antibodies, as well as human/mouse reactivity, are documented on CST, Abgent, Novus Biologicals, Biolegend and BD Biosciences websites. Antibodies were also validated in work published in Pantelidou et al., Cancer Discovery, 2019. STING KO cells demonstrated absence of bands corresponding to total and phospho-STING, as well as phospho-TBK1.

Eukaryotic cell lines

Policy information about cell lines and Sex and Gender in Research

Cell line source(s)	ATCC
Authentication	Cell line identity was verified with short tandem repeat profiling.
Mycoplasma contamination	All cell lines were routinely tested for mycoplasma contamination (Lonza LT07-318) and were negative.
Commonly misidentified lines (See ICLAC register)	No commonly misidentified lines were used in this study.

Animals and other research organisms

Policy information about studies involving animals; ARRIVE guidelines recommended for reporting animal research, and Sex and Gender in Research

Laboratory animals	Tumors were implanted into the mammary fat pad of female 6-8 week-old FVB/129P2 or NSG mice (NOD-Prkdcem26Cd52Il2rgem26Cd22/NjuCrl).
Wild animals	The study did not involve wild animals.
Reporting on sex	Only female mice were used in these experiments based on models in which tumor is orthtopically implanted in mammary fat pads.
Field-collected samples	No samples were collected from the field.
Ethics oversight	All animal experiments were approved by the Dana-Farber Cancer Institute Institutional Animal Care and Use Committee (IACUC) and conducted in accordance with IACUC-approved protocol 17-032.

Note that full information on the approval of the study protocol must also be provided in the manuscript.

Flow Cytometry

Plots

Confirm that:

☒ The axis labels state the marker and fluorochrome used (e.g. CD4-FITC).

☒ The axis scales are clearly visible. Include numbers along axes only for bottom left plot of group (a 'group' is an analysis of identical markers).

☒ All plots are contour plots with outliers or pseudocolor plots.

☒ A numerical value for number of cells or percentage (with statistics) is provided.

Methodology

Sample preparation	Mouse tumors were extracted, finely minced, blended with the gentleMACS Dissociator (Miltenyi Biotec), and digested with the MACS Miltenyi Tumor Dissociation Kit (Miltenyi Biotec #130-096-730) according to the manufacturer's instructions. Dissociated tumor cells were washed with RPMI-1640 medium and lysed with RBC Lysis Solution (Qiagen). Cells were resuspended in FACS buffer: PBS (Life Technologies) containing 0.5% BSA and 2 mmol/L EDTA (Sigma-Aldrich). The Zombie Aqua Fixable Viability Kit was applied to cells in combination with anti-mouse CD16/CD32 Fcγ receptor II/III blocking antibody (Affymetrix #14-0161-85) for 20 minutes at room temperature, prior to incubation with primary antibodies for 1 hour at 4°C. Cells were fixed and permeabilized using the FOXP3/Transcription Factor Staining Buffer Set (Affymetrix #00-5523-00), according to the manufacturer's guidelines, and incubated with antibodies for intracellular antigens overnight at 4°C. The next day, cells were washed, resuspended in PBS. and subjected to flow cytometry (see Pantelidou et al., Cancer Discovery, 2019).
Instrument	BD LSRFortessa flow cytometer
Software	Data acquisition was performed using BD FACSDiva software, and data analysis using FlowJo V10. Compensation was performed manually on FlowJo V10 software using single color and isotype controls. Signal threshold definition was defined using all-stain, unstained, and isotype controls.
Cell population abundance	At least 300,000 events were acquired per sample to allow for accurate quantification of rare populations. Minimum cell population abundance was 1% of total viable cells.
Gating strategy	The gating strategy used in the flow cytometric analysis of immune cell subsets is shown in Supplementary 4b. Debris was excluded on SSC vs FSC plot and zombie aqua-negative, i.e., viable cells were gated. Live cells were analyzed for expression of CD45 (hematopoietic cells), CD3 (total T cells), CD8 T-cells, CD4 T-cells and dual expression of CD11B/CD11C. CD8+ T-cells were further analyzed for expression of Granzyme-B and median fluorescence intensity (MFI) was derived. CD4 T-cells were analyzed for expression of Tbet and FoxP3. CD11C+CD11Bdendritic cells were analyzed for expression of CD40 and MHCII.

☒ Tick this box to confirm that a figure exemplifying the gating strategy is provided in the Supplementary Information.
